# Rational design of boron-dipyrromethene (BODIPY) reporter dyes for cucurbit[7]uril

**DOI:** 10.3762/bjoc.14.171

**Published:** 2018-07-30

**Authors:** Mohammad A Alnajjar, Jürgen Bartelmeß, Robert Hein, Pichandi Ashokkumar, Mohamed Nilam, Werner M Nau, Knut Rurack, Andreas Hennig

**Affiliations:** 1 Department of Life Sciences and Chemistry, Jacobs University Bremen, Campus Ring 1, 28759 Bremen, Germany; 2 Chemical and Optical Sensing Division, Bundesanstalt für Materialforschung und -prüfung (BAM), Richard-Willstätter-Str. 11, 12489 Berlin, Germany; 3 Laboratory of Bioimaging and Pathology, UMR 7021 CNRS, Faculty of Pharmacy, University of Strasbourg, 74 Route du Rhin, F-67401 Illkirch-Graffenstaden, France

**Keywords:** BODIPY, cucurbituril, fluorescence, pH, photoinduced electron transfer, supramolecular chemistry

## Abstract

We introduce herein boron-dipyrromethene (BODIPY) dyes as a new class of fluorophores for the design of reporter dyes for supramolecular host–guest complex formation with cucurbit[7]uril (CB7). The BODIPYs contain a protonatable aniline nitrogen in the *meso*-position of the BODIPY chromophore, which was functionalized with known binding motifs for CB7. The unprotonated dyes show low fluorescence due to photoinduced electron transfer (PET), whereas the protonated dyes are highly fluorescent. Encapsulation of the binding motif inside CB7 positions the aniline nitrogen at the carbonyl rim of CB7, which affects the p*K*_a_ value, and leads to a host-induced protonation and thus to a fluorescence increase. The possibility to tune binding affinities and p*K*_a_ values is demonstrated and it is shown that, in combination with the beneficial photophysical properties of BODIPYs, several new applications of host–dye reporter pairs can be implemented. This includes indicator displacement assays with favourable absorption and emission wavelengths in the visible spectral region, fluorescence correlation spectroscopy, and noncovalent surface functionalization with fluorophores.

## Introduction

Cucurbit[*n*]urils (CBn, *n* = 5–8, 10, and 14) are a class of macrocyclic host molecules which are water soluble, nontoxic, and are able to bind a large variety of neutral and cationic guests in their inner cavity with high affinity [1–4]. This unique combination of properties has enabled numerous applications in the life sciences, for example, for protein binding [5–6], stabilization [7], immobilisation [8], isolation [9], self-assembly [10–11], and regulation [12], or for drug solubilisation and delivery [13–15].

The combination of CBs with fluorescent dyes directly enables (bio)sensing applications through the indicator displacement principle [16–17]. Therein, the fluorescence properties of a dye are altered when encapsulated by the host, and when a competitive binder displaces the dye from the cavity, the properties of the non-encapsulated dye are regenerated. This principle has enabled, for example, real-time monitoring of enzymatic activity [18–20], the detection of membrane-transport activity [21] and membrane fusion [22], and even cellular imaging appears to be a potential future prospect [23–24].

However, most combinations of macrocyclic hosts and dyes that have so far been reported [16] are only of limited use for these currently emerging life science applications of CBs. Many of the fluorescent dyes which bind to CBs with significant fluorescence changes have a limited photostability, in particular under intense laser light illumination in confocal laser scanning microscopy [23,25], or absorb at shorter wavelengths, where biological samples show a high background from autofluorescence [26–27]. An ideal fluorescent dye would be highly photostable in biological media, have long-wavelength absorption to minimize background fluorescence from biological samples, and it would have a high fluorescence quantum yield in either bound or unbound state with a large difference in fluorescence intensity between both. In addition, a tuneable hydrophobicity to render the dye–CB complex membrane permeable or not, and a tuneable affinity for the macrocycle would be desirable.

One possibility is the utilization of monofunctionalized CBs with outer cavity-attached fluorescent dyes [22,24]. This principally allows for the modular construction of various Förster resonance energy transfer (FRET) pairs as demonstrated with a Cy3-attached CB7, or the design of self-inclusion complexes, in which an outer cavity-attached rhodamine was intramolecularly bound in the CB7 cavity. As an alternative, it has been previously suggested that host-assisted protonation of a cavity-binding functional moiety (an “anchor group”) and a suitably attached protonation-sensitive fluorescent dye yields a rational and modular approach towards CB–dye pairs [25]. This strategy had been previously applied to carbazole, aminonaphthalenesulfonate and aminopyrene as fluorescent dyes [25–28].

**Figure 1:**
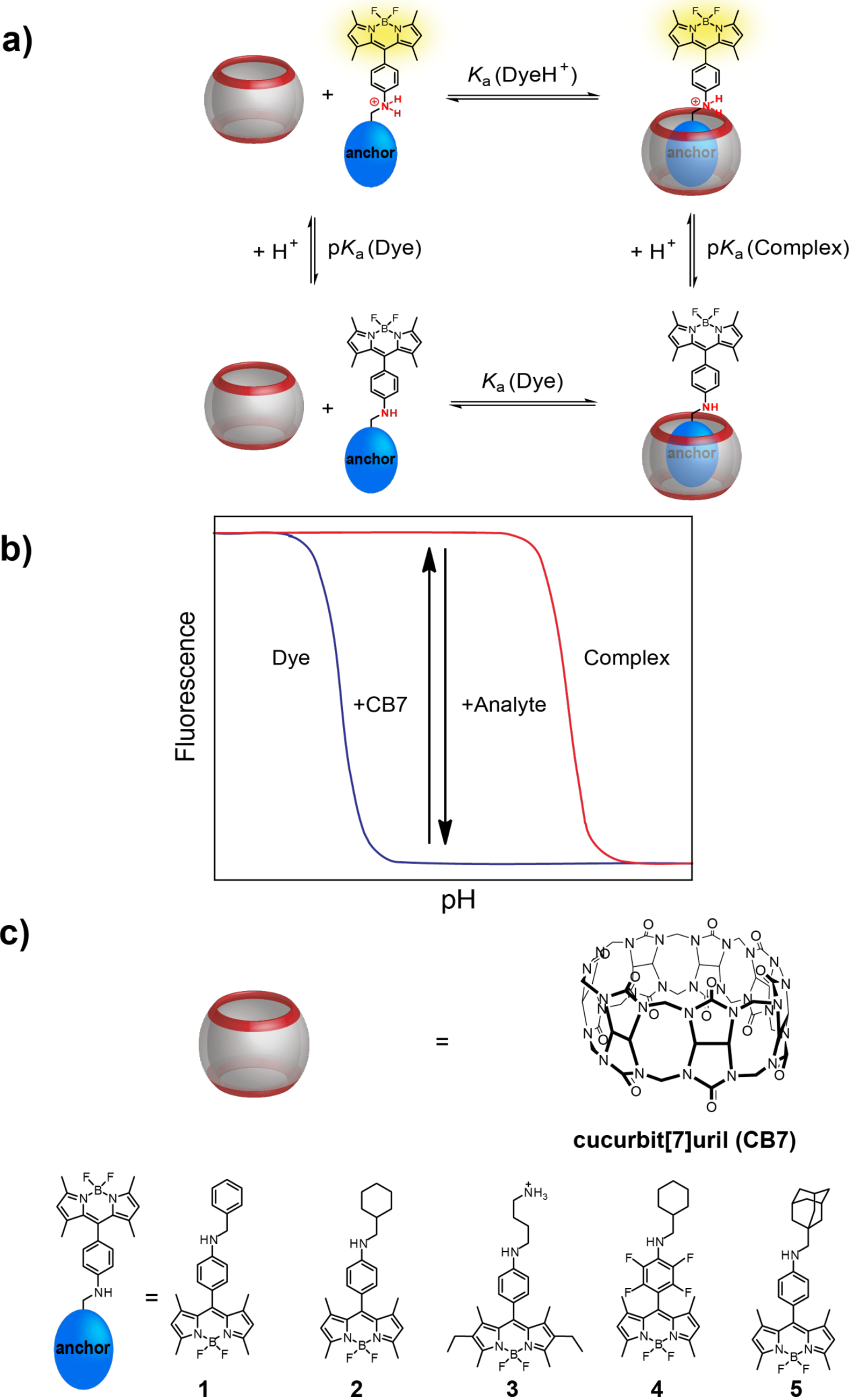
a) The “anchor group” approach for a rational design of CB–dye pairs involving a thermodynamic cycle of protonation and binding. b) Simulated pH titration curves of dye (blue) and CB7–dye complex (red) demonstrating the sensing principle based on the p*K*_a_ of the dye and the complex in the presence and absence of analyte. c) Structures of CB7 and BODIPY derivatives.

Herein, we systematically explore the utility of boron-dipyrromethenes (BODIPYs) with an aniline substituent in the *meso*-position as fluorescent dyes in this type of anchor approach (Figure 1). BODIPYs are a class of fluorescent dyes that are particularly suitable for applications in medical imaging, and as fluorescent labels in biology, biochemistry and related fields [29–30]. They are characterized by narrow absorption and fluorescence emission bands with small Stokes shifts, high molar absorption coefficients, and high quantum yields. Their excitation and emission maxima are in the visible region, usually above 470 nm, and they show high thermal and photochemical stability under various conditions, particularly under physiological conditions. Although most BODIPYs are insensitive to pH changes, pH-activatable optical probes for cancer imaging have been reported, in which an aniline substituent in the *meso*-position of the BODIPY core led to efficient fluorescence quenching by photoinduced electron transfer (PET), whereas the protonated form was brightly fluorescent [31]. We report herein the synthesis and photophysical characterization of BODIPY derivatives with an aniline substituent in the *meso*-position to which different anchor groups have been attached, and we investigate their complexation behaviour with CB7. The goal was to explore the suitability of this approach, the possibility to fine-tune binding constants with different anchor groups and to provide BODIPYs with different absorption and emission wavelengths as well as p*K*_a_ values of the aniline substituent.

## Results and Discussion

### Synthesis

**Scheme 1:**
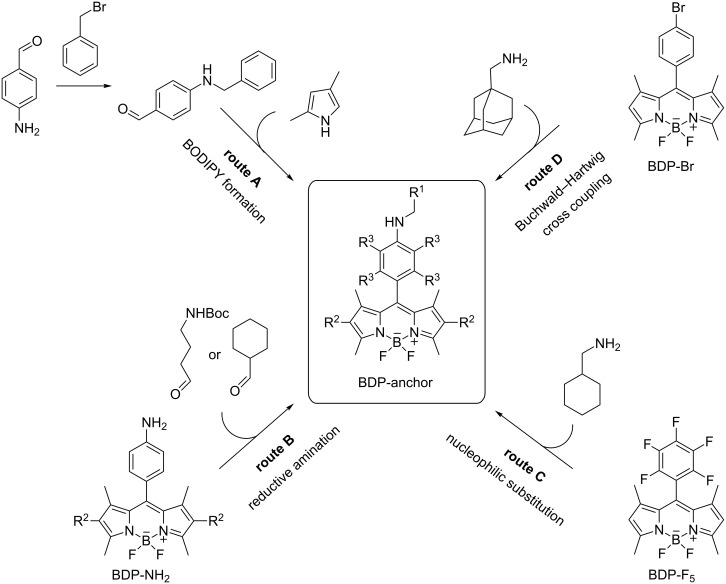
Synthesis of BODIPY derivatives.

In this paper, various routes were explored to synthesize the desired BODIPY dyes bearing an anchor group for binding to CB7 (Scheme 1). **1** was obtained by alkylation of *p*-aminobenzaldehyde with benzyl bromide and subsequent reaction of the obtained 4-(benzylamino)benzaldehyde with 2,4-dimethylpyrrole to afford the BODIPY dye by condensation under acidic conditions (route A) [32–33]. Since all efforts to obtain **2** via route A were not successful, even using Finkelstein conditions in aprotic solvents with high boiling points with various bases [34–35], BDP-NH_2_ was synthesized according to a reported literature procedure [32], and then converted into the desired BODIPY anchor dye by reductive amination with the respective aldehyde using sodium triacetoxyborohydride as a mild reducing agent (route B) [36]. **3** was also synthesized by reductive amination by reacting BDP-NH_2_ with 4-[*N*-(*tert*-butyloxycarbonyl)]amino-1-butanal [37] followed by Boc deprotection with TFA. **4** was prepared by a substitution reaction from the parent *meso*-pentafluorobenzyl-BODIPY BDP-F_5_ with aminomethylcyclohexane (route C), following an established synthetic approach [38]. For the preparation of the aminomethyladamantane derivative **5**, a route via a bromophenyl-BODIPY BDP-Br followed by a Buchwald–Hartwig coupling was performed. For the latter, a previously published Pd/XPhos containing catalytic system was successfully utilized (route D) [39].

### Spectroscopic characterization of dyes

To ensure that the dyes do not aggregate under the conditions used for further measurements, concentration-dependent absorption and fluorescence spectra were measured first. In neutral water containing either 5% or 30% (v/v) acetonitrile (ACN), the aniline nitrogen in the *meso*-position of all BODIPY dyes is unprotonated (see below) and with 30% ACN, a linear dependence of the fluorescence intensity on the concentration of the dyes with no significant alterations of the shape of the absorption and emission bands was observed over the whole range of concentrations used herein (up to 5 µM). In 5% ACN, however, dye aggregation was indicated by a downward curvature in the fluorescence intensity plots at dye concentrations above 60 to 120 nM. Further experiments were therefore conducted in 30% ACN.

**Figure 2:**
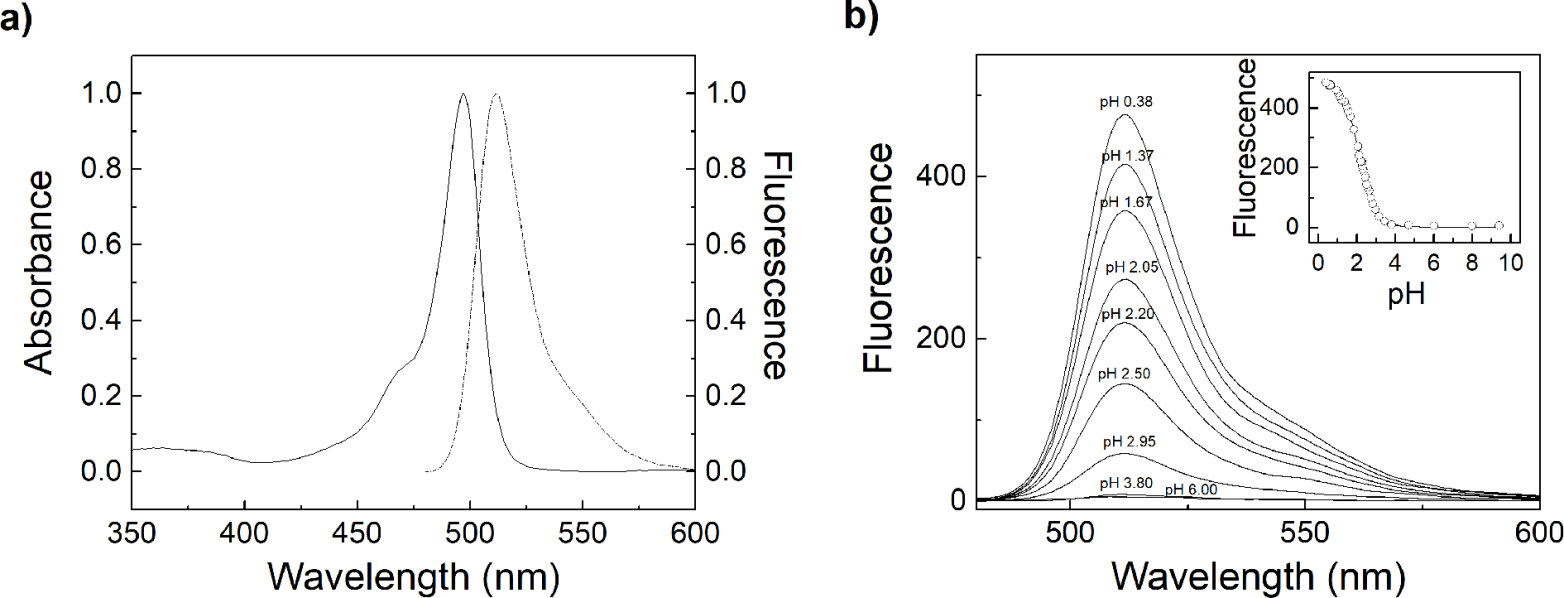
a) Normalized absorption (solid line) and normalized fluorescence emission spectrum (dotted line) of 0.72 µM **1** in 30% (v/v) ACN in water, pH 7.0, and b) fluorescence spectra in 30% (v/v) ACN in water with varying pH. Inset: Fluorescence pH titration measured with λ_exc_ = 470 nm and λ_em_ = 510 nm.

The absorption maximum was centred at ca. 500 nm for all aniline dyes (Figure 2a and Table 1) and the emission maximum was centred at ca. 510 nm for **1** and **2**, whereas **3** showed a significantly red-shifted emission maximum at 540 nm, because we used the hexaalkylated instead of the tetraalkylated BODIPY core for this dye, trying to achieve maximum fluorescence output. The spectra of the tetrafluorinated BODIPY **4** showed an overall red shift with the absorption maximum at 510 nm and the emission maximum at 530 nm. The molar absorption coefficients of the BODIPY derivatives were around 90,000 M^−1^cm^−1^, which agrees well with related BODIPY derivatives in the literature [30,40–44].

**Table 1:** Photophysical properties of the synthesized BODIPY derivatives.^a^

	**1**	**2**	**3**	**4**	**5**

ε [M^−1^cm^−1^]	97,000	97,000	93,000	85,300	99,000
λ_abs.max_ [nm]	496	497	500	510	497
λ_em.max_ [nm]	511	510	540	530	510
Φ_f_ (Dye) [%]	1.1	6.2	0.017	2.0	2.4
Φ_f_ (DyeH^+^) [%]	54	51	0.12	41^b^	30.5
p*K*_a_ (Dye)	2.2	2.6	2.7	−0.3	3.6

^a^Measured in 30% (v/v) ACN in water except for the molar absorption coefficient ε, which was determined in neat ACN. ^b^Determined from the CB7 complex at 4 mM CB7. Note that the fluorescence quantum yields of the dyes are not affected by complexation (see text for details).

With decreasing pH, a strong increase in fluorescence was observed for all dyes (Figure 2b), which is due to the protonation of the aniline nitrogen in the *meso*-position of the BODIPY core lowering the HOMO energy level of the aniline group. Negligible changes in absorption spectra and in the position of the emission maxima were in accordance with the anticipated PET mechanism [31,45]. Further, the change in free energy, ∆*G*, associated with PET was calculated using the Rehm–Weller equation [46]. Therefore, we used a reduction potential of −1.55 V for the 1,3,7,9-tetramethyl-BODIPY core acceptor of **1**, **2**, and **5** [47] and of −1.81 V for the 2,8-diethyl-1,3,7,9-tetramethyl-BODIPY core acceptor of **3** in acetonitrile [47], an oxidation potential of +0.0625 V for the aniline donor [48–49], and the vibrational zero electronic energy was determined as 2.46 eV from absorption and emission spectra. This gave ∆*G* values of −87.6 kJ mol^−1^ for **1**, **2**, and **5** and of −62.5 kJ mol^−1^ for **3**, which clearly demonstrates that PET is energetically favourable.

Fitting of the pH titration curves revealed p*K*_a_ values in the range of 2–3 for the aniline nitrogen and a p*K*_a_ value of −0.14 for the tetrafluoroaniline nitrogen of BODIPYs (Table 1). This range agrees well with the electron-withdrawing nature of the BODIPY core and with reported p*K*_a_ values, for example, for aniline (p*K*_a_ = 4.58), 4-nitroaniline (p*K*_a_ = 1.02), 4-cyanoaniline (p*K*_a_ = 1.74), or pentafluoroaniline (p*K*_a_ = −0.30) [50–51]. At basic pH values, no spectroscopic changes were noted except for **3**, which showed a broadening and a marked decrease of the absorption band (Figure S17, Supporting Information File 1). This presumably originates from a deprotonation of the terminal alkylammonium group of the putrescine chain, which could fold back and enable an intramolecular charge transfer state of the amine lone pair with the BODIPY chromophore. In accordance with this hypothesis, a positive solvatochromism with varying contents of ACN was observed (Figure S18, Supporting Information File 1).

The fluorescence quantum yields of the unprotonated BODIPY dyes were determined in 30% (v/v) ACN in water (at pH 7.4) and of the protonated BODIPY dyes in 30% ACN in 0.1 M HCl. For both, fluorescein in 0.1 M NaOH was used as the reference (Φ_f_ = 0.89) [52]. These measurements revealed an increase in fluorescence by a factor of 7 to 50 upon protonation for the investigated BODIPYs, which is sufficient for the desired sensing applications (Table 1) [20,53]. Surprisingly, and despite the hexaalkylated core was used, the fluorescence quantum yields of protonated as well as unprotonated **3** were more than 100-fold lower than the quantum yields of the other derivatives. Such reduced quantum yields have been previously reported for some BODIPYs substituted with diamines in the aniline *meso*-position, and the decreased quantum yields were ascribed to the loose-bolt effect [41,54–55].

### Complexation with CB7

**Figure 3:**
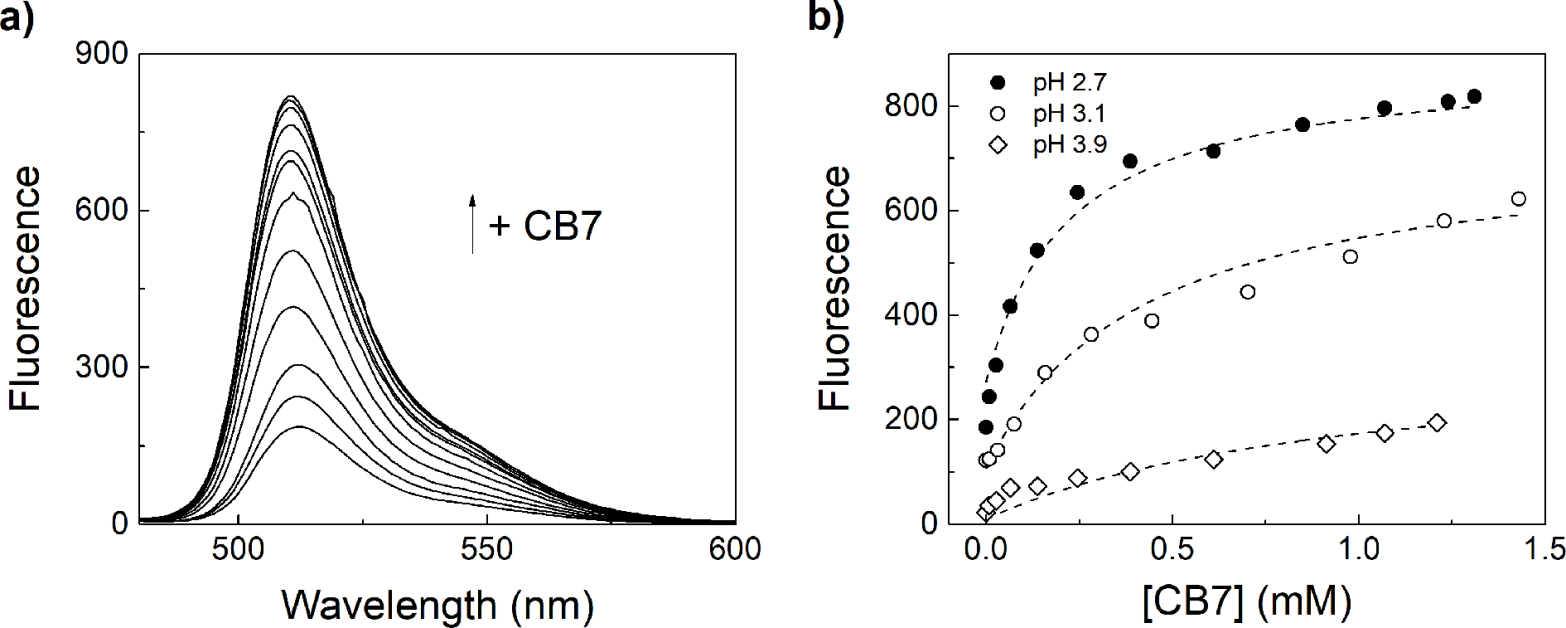
a) Fluorescence spectral changes (λ_exc_ = 470 nm) upon addition of CB7 to 50 nM **1** in 10 mM citrate buffer, pH 2.7, containing 30% (v/v) ACN in water, and b) respective titration plot (λ_em_ = 510 nm) at varying pH. The dashed lines were obtained by a global fitting according to the thermodynamic cycle in Figure 1 (see Supporting Information File 1 for details).

Addition of excess CB7 to the BODIPY dyes at low pH values, in which the dyes are fully protonated, or at high pH values above the p*K*_a_ value of the BODIPY•CB7 complex (see below) had no effect on the spectroscopic properties of the dyes. For example, the fluorescence quantum yield of **2** was identical in absence and presence of CB7 at pH 1.5. At intermediate pH values, however, the fluorescence of the dyes increased upon addition of CB7 (Figure 3). This result is in accordance with the anticipated anchor group mechanism leading to a complexation-induced protonation of the dye (Figure 1). It also suggests that the BODIPY core is not encapsulated in the macrocyclic cavity and that encapsulation of the anchor group by CB7 has no effect on the spectroscopic properties of the dyes. At intermediate pH, the protonated fraction of the dye will be strongly bound by CB7, which affects the protonation equilibrium of the dye and leads to more protonated dye being produced. The net outcome is an increase in fluorescence intensity (upward arrow in “detection window” in Figure 1b). Multiple binding titrations performed at different pH values were fully consistent with the mechanism (Figure 3b). At low pH values, the fluorescence intensity in absence of CB7 was higher and the fluorescence increase upon addition of CB7 was steeper, because more dye molecules are already protonated, whereas at higher pH values more CB7 was required to reach the final fluorescence intensity of the fully protonated dyes.

Unfortunately, the absence of any detectable changes for the fully protonated or unprotonated dyes upon addition of CB7 prevented a direct determination of the respective binding constants, *K*_a_(DyeH^+^) and *K*_a_(DyeH), at low and high pH values. We therefore developed a global fitting procedure (see Supporting Information File 1), in which the binding titrations at different pH values are simultaneously analysed to provide the values for the binding constants of the protonated and unprotonated dye, *K*_a_(DyeH^+^) and *K*_a_(Dye), as well as the p*K*_a_ values of the BODIPY•CB7 complex p*K*_a_(Complex), see Table 2. The p*K*_a_ value of the uncomplexed dye, p*K*_a_(Dye), was obtained from a simple pH titration and fixed during the global fitting procedure.

**Table 2:** Properties of the CB7–BODIPY host–guest complexes.^a^

	**1**	**2**	**4**	**5**

p*K*_a_(Complex)^b^	5.0	5.3	1.5	8.2
Δp*K*_a_	2.8	2.7	1.8	4.6
*K*_a_(Dye)^c^ [M^−1^]	30	5000	240	n.a.^d^
*K*_a_(DyeH^+^)^c^ [M^−1^]	1.9 × 10^4^	2.6 × 10^6^	1.5 × 10^4^	n.a.^d^

^a^Measured in 30% (v/v) ACN/H_2_O. ^b^Error ±0.2 p*K*_a_ units. ^c^Error in *K*_a_ ca. 20%. ^d^Binding constants could not be determined due to the very slow exchange kinetics of the 5•CB7 complex, see also Figure S19 (Supporting Information File 1).

The binding affinities of the BODIPY dyes were significantly lower than the reported binding constants of the respective anchor groups in water [4]. To allow a better comparison, we determined the binding constants of the benzylammonium (Bnz) and cyclohexylmethylammonium (cyH) cations by displacement titrations (see below) in our mixture of 30% (v/v) ACN/H_2_O, which gave *K*_a_(Bnz) = 1.4 × 10^5^ M^−1^ and *K*_a_(cyH) = 1.5 × 10^7^ M^−1^. This indicated that the binding affinity is lowered 100 to 1000-fold by reducing the hydrophobic effect in presence of 30% acetonitrile as also previously noted for water/DMSO mixtures [56]. The attachment of the BODIPY chromophore to the anchor groups thus reduces the binding constant by an additional factor of 10 for the aniline *meso-*group and by a factor of 1000 for the tetrafluoroaniline group in **4**. We ascribe this to steric hindrance between the carbonyl-fringed CB7 rim and the fluorine atoms in the tetrafluoroaniline, which are slightly larger than the hydrogen atoms [57]. The data obtained with **3** could not be fitted satisfactorily, which is presumably due to the more complex photophysics of this dye (see above) and the exchange of **5** was too slow to equilibrate during the titration within reasonable time (Figure S19, Supporting Information File 1).

**Figure 4:**
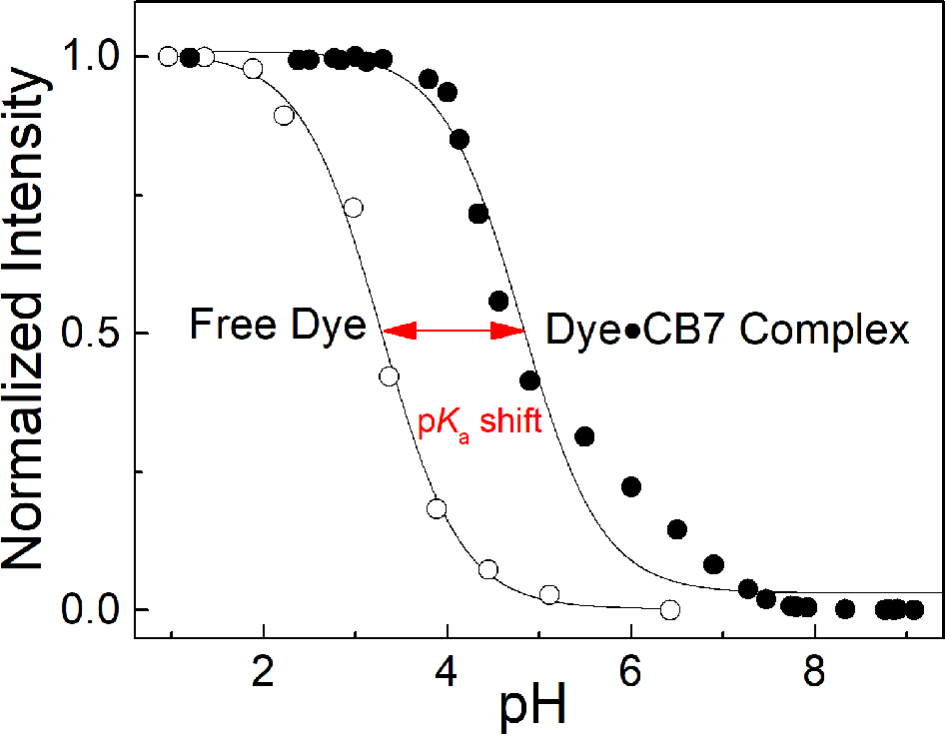
Fluorescence pH titration of **2** and the respective complex (in presence of 3 mM CB7) in 30% (v/v) ACN in water with varying pH. Fluorescence was measured with λ_exc_ = 470 nm and λ_em_ = 510 nm.

The p*K*_a_ values of the host–dye complex were independently determined by pH titrations in presence of excess CB7 and analysed assuming quantitative complex formation (Figure 4). Overall, the p*K*_a_ values from the direct titration and from the global fitting agreed reasonably well, and the complexation-induced p*K*_a_ shifts were in the typical range reported for CB7 host–guest complexes [58].

### Application of BODIPY-CB7 complexes

**Figure 5:**
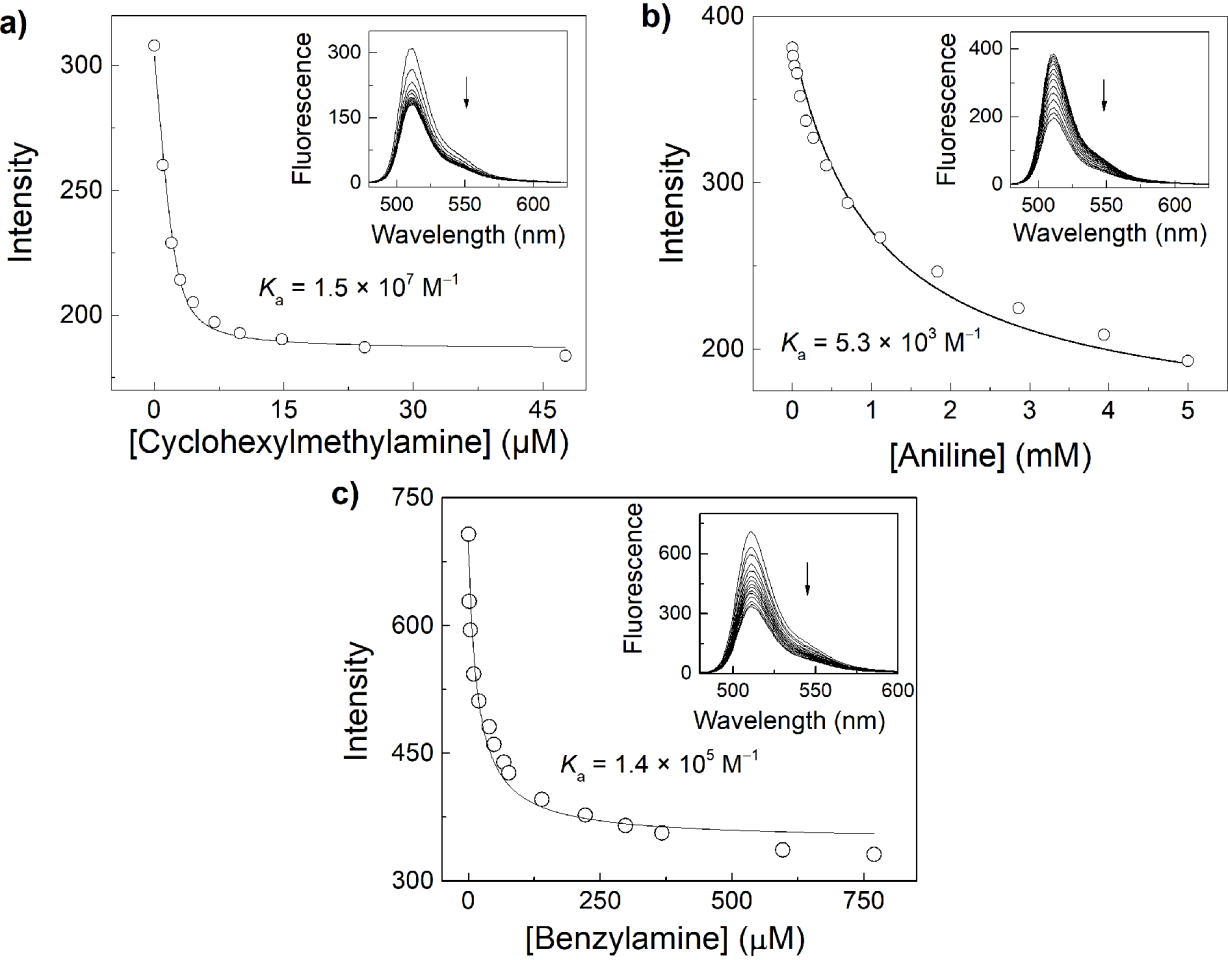
Fluorescence displacement titrations (λ_ex_ = 470 nm, λ_em_ = 510 nm). a) 5 µM **2** and 2.5 µM CB7 with cyclohexylmethylamine. b) 7 µM **2** and 2.5 µM CB7 with aniline. c) 0.5 µM **2** and 0.2 µM CB7 with benzylamine in 10 mM citrate buffer in 30% (v/v) ACN in water, pH 3.1.

The availability of BODIPY dyes, which respond towards complexation by CB7, enables a large variety of potential applications of the resulting host–dye reporter pairs. As first example, the CB7–BODIPY pairs can be applied as sensors using the indicator displacement principle [17–18,21,59]. This is demonstrated by sensing of cyclohexylmethylamine and aniline as model analytes (Figure 5). In order to determine the binding constants of the two analytes, the apparent binding constant of **2** at pH 3.1 was taken (*K*_app_ = 6.7 × 10^5^ M^−1^) and the displacement titrations were analysed with a competitive titration model [18,25]. This gave binding constants of 1.5 × 10^7^ M^−1^ for the cyclohexylmethylammonium cation, 5.3 × 10^3^ M^−1^ for the anilinium cation and 1.4 × 10^5^ M^−1^ for the benzylammonium cation in the 10 mM citrate buffer in 30% (v/v) ACN in water.

**Figure 6:**
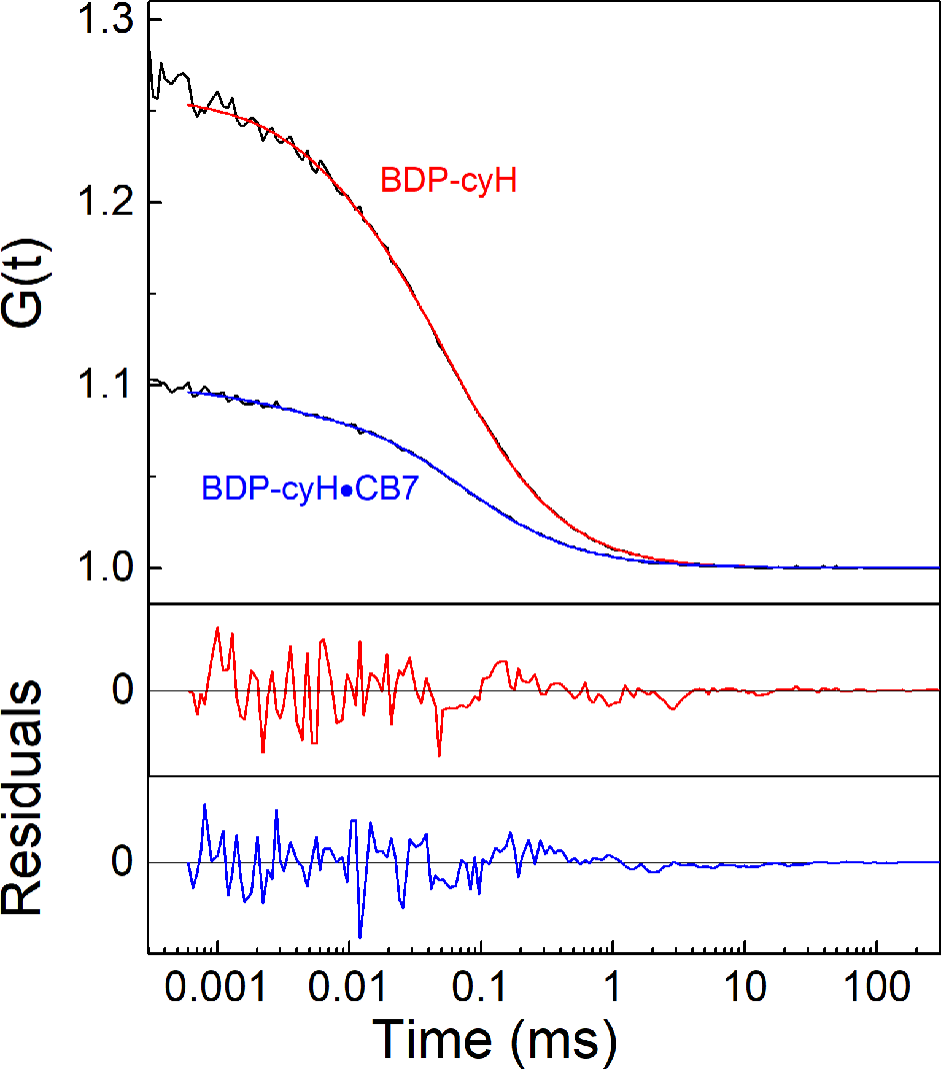
FCS autocorrelation curves obtained with 10 nM **2** in the absence (red fitted line) and presence (blue fitted line) of 100 µM CB7 at pH 1.5 in 30% (v/v) ACN in water. The fitted diffusion times for the free dye and the complex were 54.1 and 77.9 µs, respectively.

As another advantage over previously established supramolecular reporter dyes, the absorption maximum of the BODIPYs introduced herein matches the emission wavelength of an Ar laser, which is still the most common excitation source in fluorescence correlation spectroscopy (FCS) and fluorescence microscopy. FCS has been established to study dynamic processes in biological systems and, more recently, also in materials science, but its use in supramolecular chemistry is so far very rare [60–64]. It can be applied to investigate translational and rotational diffusion of supramolecules as well as exchange kinetics. To demonstrate the compatibility of the new BODIPY dyes with FCS, we have determined the diffusion coefficient of the **2**•CB7 complex in comparison to the free **2** dye. FCS autocorrelation curves (Figure 6) were analysed to obtain the diffusion times *t*_diff_ of **2** and the **2**•CB7 complex and then converted into diffusion coefficients *D* using the reported standard rhodamine 6G (*D* = 2.80 × 10^−6^ cm^2^ s^−1^) [60,65]. This gave *D* = 4.87 × 10^−6^ cm^2^ s^−1^ for **2** and *D* = 3.39 × 10^−6^ cm^2^ s^−1^ for the **2**•CB7 complex, which perfectly matches the range reported for other dyes and their respective CB7 complexes [60]. In accordance with inclusion of the anchor group into the CB7 cavity and thus exclusion complex formation of the BODIPY core, the photostability of the dyes was not affected by CB7 complex formation (Figure S20, Supporting Information File 1).

**Figure 7:**
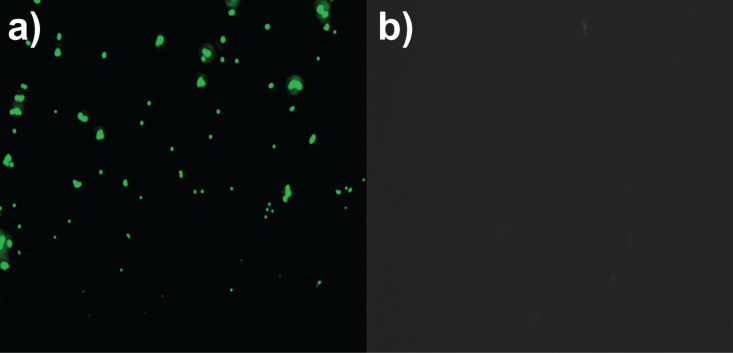
Fluorescence microscopy images of 1 mg/mL polymer microspheres a) with or b) without surface-bound CB7 after incubation with 10 nM **5** and 1 µM AMADA in 10 mM citrate, pH 3.3 (30% (v/v) ACN in water) and centrifugation to immobilize **5** through supramolecular host–guest binding.

The compatibility of BODIPYs with common excitation sources and filter sets also enables their use in fluorescence microscopy. To demonstrate, we have used polymer microparticles with surface-bound CB7 [66] and added them to a solution containing a mixture of **5** and 1-(aminomethyl)adamantane (AMADA). The latter was added to reduce the surface group density of the dye and prevent undesired self-quenching at high surface concentrations of the fluorophore. After centrifugation and washing of the polymer particles, surface-bound **5** could be clearly visualized by fluorescence microscopy on CB7-functionalized polymer particles, whereas polymer particles lacking CB7 on the surface did not show any fluorescence (Figure 7). This result is consistent with specific host–guest binding of **5** to CB7 on the surface, which suggests the use of **5** for straightforward surface functionalization to create nanophotonic devices as well as for multimodal surface group quantifications, e.g., using their optical properties for fluorescence and their fluorine heteroatom for X-ray photoelectron spectroscopy [67–69].

## Conclusion

We have established herein BODIPYs as fluorophores in the anchor group strategy towards the design of reporter dyes for CB7. The resulting dyes have absorption and emission wavelengths which are compatible with established instrumentation in life science applications and show pronounced fluorescence changes upon host binding. The affinity of the dyes for the CB7 host was successfully adjusted by using different anchor groups and was only minimally reduced in comparison with the unmodified anchor groups. This strategy enables several applications of fluorescent host–guest complexes, for example, indicator displacement assays with absorption and emission wavelengths in the visible spectral region, fluorescence correlation spectroscopy, and noncovalent surface functionalization with fluorophores. Furthermore, the strategy is similarly applicable to pH-sensitive fluoresceins, cyanines or rhodamines, in which protonation and deprotonation of suitably positioned amino groups can also modulate their fluorescence properties. It can also be used to design dyes which reduce their fluorescence upon binding, e.g., when electron-poor groups are generated by protonation which are quenched intramolecularly by donor-excited PET [70–74].

## Supporting Information

File 1 Experimental details and supporting figures.
