# Supplementary material for: Rational design of boron-dipyrromethene (BODIPY) reporter dyes for cucurbit[7]uril
Source: Beilstein J Org Chem. 2018 Jul 30;14:1961–71. doi: 10.3762/bjoc.14.171 (PMC6122296; doi:10.3762/bjoc.14.171)
Supplement: File 1 — Experimental details and supporting figures. [file Beilstein_J_Org_Chem-14-1961-s001.pdf]

Supporting Information

for

Rational design of boron-dipyrromethene (BODIPY) reporter dyes for cucurbit[7]uril

Mohammad A. Alnajjar[1], Jürgen Bartelmeß[2], Robert Hein[1], Pichandi Ashokkumar[2,3], Mohamed Nilam[1], Werner M. Nau[1], Knut Rurack[2] and Andreas Hennig*,[1]

Address: [1]Department of Life Sciences and Chemistry, Jacobs University Bremen, Campus Ring 1, 28759 Bremen, Germany, [2]Chemical and Optical Sensing Division, Bundesanstalt für Materialforschung und -prüfung (BAM), Richard-Willstätter-Str. 11, 12489 Berlin, Germany and [3]Present address: Laboratory of Bioimaging and Pathology, UMR 7021 CNRS, Faculty of Pharmacy, University of Strasbourg, 74 Route du Rhin, F-67401 Illkirch-Graffenstaden, France

Email: Andreas Hennig - a.hennig@jacobs-university.de

* Corresponding author

Experimental details and supporting figures

Table of Contents

1 Materials and instrumentation S2
2 Abbreviations S3
3 Synthesis S4
3.1 Synthesis of BDP-cyH (**2**) S4
3.2 Synthesis of BDP-Bnz (**1**) S5
3.3 Synthesis of BDP-Put (**3**) S6
3.4 Synthesis of $BDPF_4$-cyH (**4**) S8
3.5 Synthesis of BDP-AMADA (**5**) S10
3.6 NMR Spectra of Synthesized Compounds S12
4 Determination of fluorescence quantum yields S20
5 Global fitting procedure S21
6 Supporting figures S23
7 References S25

1 MATERIALS AND INSTRUMENTATION

Materials. Reagents and compounds for synthesis, buffer preparation and analytical measurements were from Sigma-Aldrich (Steinheim, Germany). Deuterated solvents for NMR measurements were from Deutero (Kastellaun, Germany), Sigma-Aldrich (Steinheim, Germany) or Merck (Mannheim, Germany). Cucurbit[7]uril (CB7) was from Strem Chemicals Int. (Massachusetts, United States) or synthesized as previously reported [1]. Buffers were prepared from solid citric acid monohydrate and the pH was adjusted by addition of NaOH.

Instrumentation. NMR spectra were recorded on a JEOL ECX 400 spectrometer and are reported as chemical shifts (δ) in ppm relative to TMS ($\delta = 0$) or residual protonated solvent signals as internal standard. Spin multiplicities are reported as singlet (s), doublet (d), triplet (t), quartet (q), and quintet (quint) with coupling constants (*J*) given in Hz, or as multiplets (m). FTIR spectra were recorded on a Bruker IR Spectrometer VECTOR 33 and are reported as wavenumbers in cm^{-1} with band intensities indicated as s (strong), m (medium), w (weak), and br (broad). ESI-HRMS was performed on a Bruker HCT ultra mass spectrometer, and spectra are reported as mass-per-charge ratio m/z. Absorbance measurements were performed with a Varian Cary 4000 spectrophotometer. Fluorescence was measured either with a Varian Eclipse or a Jasco FP-8500 spectrofluorometer equipped with temperature controllers. Correction curves for the fluorescence measurements were generated with a dye kit certified by the Federal Institute for Materials Research and Testing (BAM, Germany) [2-6]. All spectroscopic measurements were performed in 3.5 ml quartz glass cuvettes from Hellma Analytics (Müllheim, Germany). pH values were measured with a Weilheim 3110 pH meter and the pH values in ACN/water mixtures are reported as uncorrected, apparent pH values. Fluorescence correlation spectroscopy (FCS) was performed with an Insight Cell 3D microscope from Evotec Technologies (Hamburg, Germany, now Perkin Elmer), equipped with a 543-nm continuous-wave HeNe and a 635-nm pulsed diode laser (~80-ps pulse width; PicoQuant, Berlin, Germany), and a 40fold water immersion objective (UApo340 40×, NA 1.15, Olympus, Tokyo, Japan), and avalanche photodiode detectors (SPCM-AQR-13-FC; Perkin Elmer Optoelectronics, Fremont, CA). Fluorescence excitation was

performed with linear polarized light, and the laser power was adjusted to 5–20 mW. Fluorescence microscopy images were captured by a Zeiss Axiovert 200 with a BP 455-495/LP 515 filter set through an Evolution QEi Media Cybernetics camera by using a 40x objective, and processed with the software imageJ V1.48 (https://imagej.nih.gov/ij/index.html).

2 ABBREVIATIONS

ACN: acetonitrile, AMADA: 1-(aminomethyl)adamantane, BODIPY: 4,4-difluoro-4-bora-3a,4a-diaza-s-indacene, CB7: cucurbit[7]uril, DCM: dichloromethane, DCE: dichloroethane, DDQ: 2,3-dichloro-5,6-dicyano-*p*-benzoquinone, DMF: dimethylformamide, EtOAc: ethyl acetate, EtOH: ethanol, ESI-HRMS: electrospray ionization high resolution mass spectrometry, Et_2O: diethyl ether, ESI: electrospray ionization, DiPEA: diisopropylethylamine, HOMO: highest occupied molecular orbital, FT-ICR: Fourier transform ion cyclotron resonance, FTIR: Fourier transform infrared spectroscopy, HRMS: high resolution mass spectrometry, IR: infrared spectroscopy, MeOH: methanol, MS: mass spectrometry, NIR: near-infrared, NMR: nuclear magnetic resonance spectroscopy, PE: petroleum ether 40-60 °C, PET: photoinduced electron transfer, TEA: triethylamine, TFA: trifluoroacetic acid, TMS: tetramethylsilane.

3 SYNTHESIS

3.1 Synthesis of BDP-cyH (2)

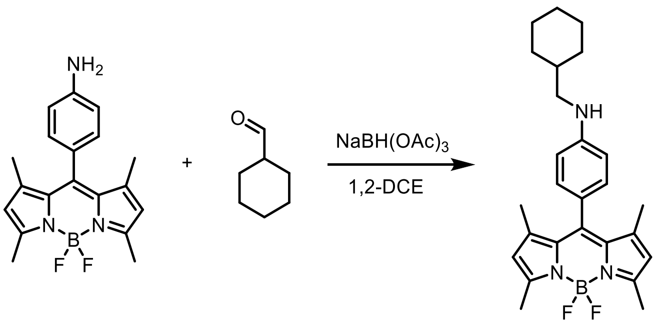

BDP-cyH (2). 69 mg $Me_4BDP\text{-}NH_2$ (0.203 mmol) and 27 µL cyclohexanecarbaldehyde (0.223 mmol) were dissolved in 5 mL 1,2-dichloroethane. Then, 60 mg $NaBH(OAc)_3$ (0.283 mmol) and 12 µL AcOH (0.210 mmol) were added and the mixture was reacted for 2 days (48 h) at room temperature under N_2. Afterwards, 1 mL of 0.1 M NaOH and then 45 mL Et_2O were added. The organic phase was washed twice with 20 mL brine and dried over anhydrous Na_2SO_4. Removal of diethylether by rotary evaporation and purification on a silica column (PE/EtOAc 10:1) gave 38 mg (43%) BDP-cyH as an orange-red product. R_f = 0.79 with PE/EtOAc (2:1). M.p 217 °C; ^{1}H NMR 400 MHz, $CDCl_3$ δ (ppm) = 6.99 (d, J = 6.2 Hz, 2H,Ar-H), 6.68 (d, J = 5.9 Hz, 2H, Ar-H), 5.96 (s, 2H, Pyr-H), 2.99 (d, J = 6.6 Hz, 2H, Cy-CH_2-NH), 2.54 (s, 6H, Pyr-CH_3), 1.88-1.67 (m,5H, cyH), 1.43 (s, 6H, Pyr-CH_3), 1.35- 0.95 (m, 6H, cyH). ^{13}C NMR (100 MHz, $CDCl_3$) δ = 154.9, 149.1, 143.4, 143.2, 132.3, 129.0, 123.2, 121.0, 113.2, 50.8, 45.0, 37.8, 31.4, 29.8, 26.7, 26.1, 14.8, 14.7. ^{19}F NMR (376 MHz, $CDCl_3$) δ = –146.0. ATR-IR cm^{-1} 3383 (w), 2924 (m), 2852 (w), 1741 (w), 1613 (w). MS (ESI, DCM): 436.2731m/z ($[M+H]^+$).

3.2 Synthesis of BDP-Bnz (1)

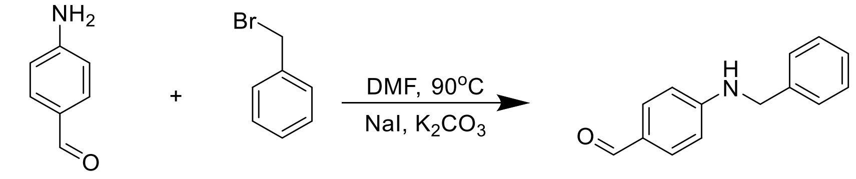

***p*-(benzylamino)benzaldehyde.** 200 mg (1.65 mmol) *p*-aminobenzaldehyde was dissolved in 40 ml of anhydrous dimethylformamide (DMF). 1.14 g (8.25 mmol) NaI and 1.14 g (8.25 mmol) K_2CO_3 was added and the mixture was stirred for 1 h at ambient temperature. 197 mg (1.65 mmol) (bromomethyl)benzene was added dropwise within 15 min and the reaction was subsequently heated to 90 ^oC for 3 d. After that, the reaction mixture was allowed to cool down to room temperature and 50 mL water were added. 40 mL DCM was added and the organic phase was separated and subsequently washed two times with 50 mL saturated brine solution. The combined organic phases were dried with $MgSO_4$ and the solvent was removed by rotary evaporation. Purification by column chromatography using a solvent gradient from PE/ EtOAc 50:1 to 1:2 (v/v) gave 170 mg (48%) *p*-(benzylamino)benzaldehyde as a yellow solid. R_f = 0.51 with PE/EtOAc (2:1). Mp: 125 °C; ^{1}H NMR (400 MHz, acetone-d_6): δ (ppm) = 9.67 (s, 1H, COH), 7.64 (d, *J* = 8.4 Hz, 2H, Ar-H), 6.77 (d, *J* = 8.4 Hz, 2H, Ar-H), 7.52-7.11 (m, 5H, Ar-H), 4.49 (2H, Ar-CH_2-NH). ^{13}C NMR (100 MHz, actone-d_6) δ (ppm) = 190.0, 154.9, 140.1, 132.6, 129.5, 128.2, 127.3, 112.8, 47.4; MS (ESI+, ACN: 234 m/z ([M+Na]$^+$).

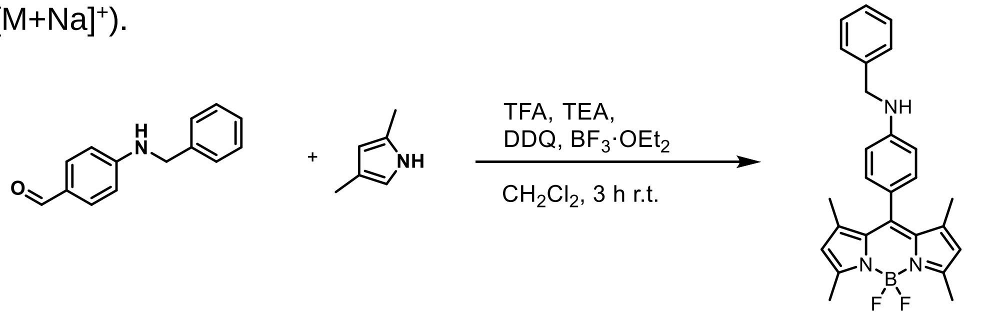

BDP-Bnz (1). 100 mg (0.47 mmol) *p*-(benzylamino)benzaldehyde were dissolved in 10 mL DCM and 97 µL 2,4-dimethylpyrrole (0.95 mmol) and 5 µL TFA were added to the reaction. After 50 min reaction at room temperature, 0.107 g (0.47 mmol) DDQ were added, the reaction mixture was stirred for another 50 min and 2 ml (14.35 mmol) TEA were added. After 30 min, 2 mL (16.2 mmol) boron trifluoride diethyl etherate were added and the

reaction mixture was stirred for 3 hours. Evaporation of the solvent gave a dark-purple solid, which was further purified by column chromatography using a gradient from PE/EtOAc 4:1 to 1:1 to afford 35 mg (17%) BDP-Bnz as a purple solid. R_f = 0.3 with PE/EtOAc (2:1). Mp: 227 °C; ^{1}H NMR 400 MHz, acetone-d6 δ (ppm) = 7.43-7.20 (m, 5H, Ar-H), 7.04 (d, *J* = 8.3 Hz, 2H, Ar-H) 6.86 (d, *J* = 8.3 Hz, 2H, Ar-H), 6.08 (s, 2H, Pyr-H), 4.44 (s, 2H, Ar-*CH₂*-NH), 2.84 (s, 6H, Pyr-*CH₃*), 1.52 (s, 6H, Pyr-*CH₃*). ^{13}C NMR (100 MHz, $CDCl_3$) δ = 155.0, 143.3, 142.8, 132.2, 129.1, 128.8, 127.7, 121.1, 113.9, 100.0, 48.6, 14.8. ^{19}F NMR (376 MHz, $CDCl_3$) δ = –146.2. ATR-IR cm^{-1} 3418 (m), 2924 (w), 2852 (w), 1738 (w), 1613 (s). MS (ESI, DCM): 430.30 m/z ($[M+H]^+$).

3.3 Synthesis of BDP-Put (3)

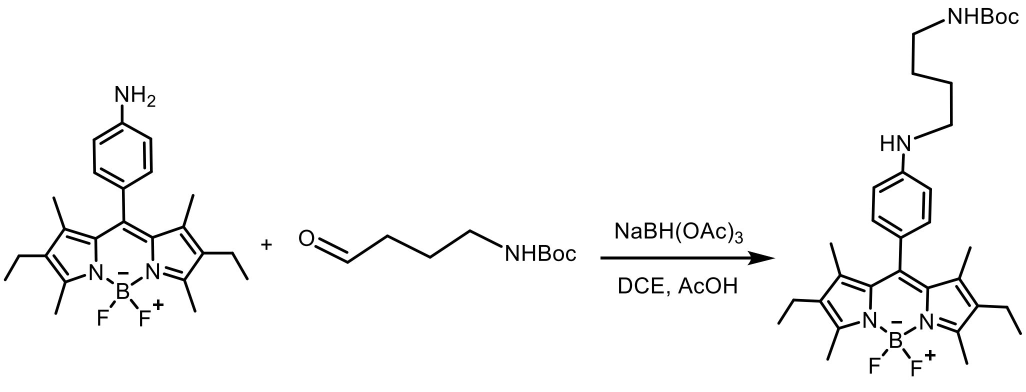

BDP-PutBoc

BDP-PutBoc. To a stirred solution of Me_4Et_2BDP-NH_2 (100 mg, 0.253 mmol) in 1,2-dichloroethane (10 mL), 4-[*N*-(*tert*-butyloxycarbonyl)]amino-1-butanal (62 mg, 0.329 mmol)[7] and AcOH (25 µL, 0.430 mmol) were added under an argon atmosphere. To this mixture, $NaBH(OAc)_3$ (70 mg, 0.329 mmol) was added in small portions over a 10 min period and stirring continued for 24 h in Ar atmosphere. After completion, the reaction was quenched by the slow addition of sat. $NaHCO_3$ (10 mL) and extracted three times with ethyl acetate (3 × 25 mL). The combined organic layers were washed with H_2O and brine (30 mL each), dried over anhydrous Na_2SO_4, filtered, and evaporated under reduced pressure. Purification on silica column (DCM/MeOH 95:5) gave 89 mg (62%) of BDP-PutBoc.^{1}H NMR (400 MHz, DMSO-d_6): δ (ppm) 7.07 (d, *J* = 8.8 Hz, 2H, Ar-H), 6.78 (d, *J* = 8.6 Hz, 2H, Ar-H), 3.15-3.28 (m, 4H, PhNH-*CH₂*-CH₂ & CH₂-*CH₂*-NHBoc), 2.38 (q, *J* = 7.2 Hz, 4H, Pyr-*CH₂*-CH₃), 2.32 (s, 6H, Pyr-*CH₃*), 1.75-1.59 (m, 4H, NHCH₂-*CH₂*-*CH₂*-

CH_2), 1.56 (s, 6H, Pyr-*CH_3*), 1.42 (s, 9H, Boc-*CH_3*), 1.04 (t, J = 7.4 Hz, 6H, Pyr-CH_2-*CH_3*).^{13}C NMR (100 MHz, DMSO-d_6): δ (ppm) 155.4, 152.5, 146.7, 141.1, 138.6, 132.8, 131.2, 130.0, 122.8, 112.3, 79.7, 53.2, 36.1, 28.6, 26.2, 25.4, 17.1, 14.6, 12.4, 11.4. HR-MS (ESI+): *m/z* calculated for $C_{32}H_{46}BF_2N_4O_2$ [M+H]$^+$: 567.3682, found 567.3678.

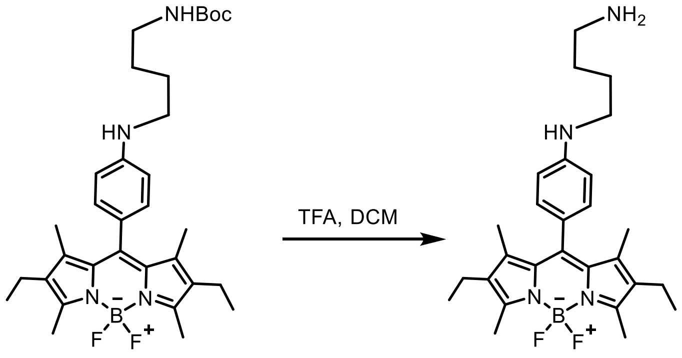

BDP-Put

BDP-Put (3). To a solution of BDP-PutBoc (80 mg, 0.141 mmol) in dichloromethane (2 mL), TFA (2 mL) was added and stirred for 3 h. The solvent was removed from the reaction mixture by rotary evaporation. Now methanol is added and evaporation continued for 3 more times to remove traces of TFA. Purification on neutral alumina column (DCM/MeOH 90:10) gave 58 mg (88%) of BDP-Put. ^{1}H NMR (400 MHz, DMSO-d_6): δ (ppm) 7.08 (d, *J* = 8.8 Hz, 2H, Ar-H), 6.79 (d, *J* = 8.4 Hz, 2H, Ar-H), 3.19 (t, *J* = 6.8 Hz, 2H, PhNH-*CH_2*-CH_2), 2.78 (t, *J* = 6.8 Hz, 2H, CH_2-*CH_2*-NH_2), 2.40 (q, *J* = 7.2 Hz, 4H, Pyr-*CH_2*-CH_3), 2.33 (s, 6H, Pyr-*CH_3*), 1.69-1.62 (m, 4H, NHCH_2-*CH_2*-*CH_2*-CH_2), 1.58 (s, 6H, Pyr-*CH_3*), 1.05 (t, *J* = 7.6 Hz, 6H, Pyr-CH_2-*CH_3*).^{13}C NMR (100 MHz, DMSO-d_6) δ = 152.7, 146.9, 140.9, 138.7, 132.8, 131.0, 130.2, 122.9, 112.4, 53.1, 41.9, 25.4, 25.1, 17.0, 14.6, 12.4, 11.4. HR-MS (ESI+): m/z calculated for $C_{27}H_{38}BF_2N_4$ [M+H]$^+$: 467.3158, found 467.3160.

3.4 Synthesis of BDPF$_4$-cyH (4)

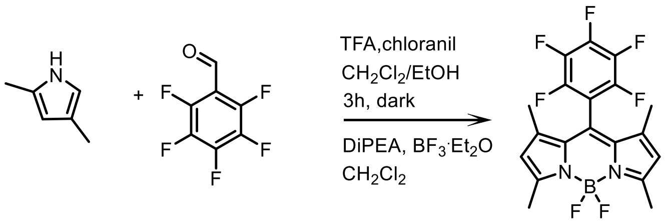

BDP-F$_5$. 0.9 g (9.4 mmol) of 2,4-dimethylpyrrole and 0.87 g (4.7 mmol) of pentafluorobenzaldehyde were dissolved in a mixture 280 mL of dichloromethane and 20 mL of absolute ethanol and deoxygenated with Ar. Some drops of trifluoroacetic acid were added and the reaction mixture was stirred for 3 h in the dark. 1125 mg (4.65 mmol) of chloranil were added, followed by stirring for 1 h. The solvents were removed and the solid was dissolved in 200 mL of dichloromethane. Then, 4.9 mL of diisopropylethylamine were added and after 5 min of stirring, 5.2 mL of boron trifluoride diethyletherate were added, followed by stirring for 30 min. The reaction mixture was filtered through a pad of silica, eluted with dichloromethane and the solvents were evaporated to dryness. The crude product was purified on a silica column (PE/DCM 2:1 with increasing amounts of DCM). After recrystallization from DCM layered with methanol and washing of the crystals with a small amount of methanol, 561 mg (1.35 mmol, 19%) of a crystalline red solid were recovered. ^{1}H NMR 400 MHz, DMSO-d$_6$ δ (ppm) = 6.30 (s, 2H, Pyr-H), 2.48 (s, 6H, Pyr-*CH$_3$*), 1.64 (s, 6H, Pyr-*CH$_3$*). ^{13}C NMR (100 MHz, DMSO-d$_6$) δ = 157.5, 144.4, 142.1, 130.1, 122.5, 14.3, 13.2. HRMS-ESI: m/z: calculated for $C_{19}H_{15}BF_7N_2^+$: 415.1211 [M+H]$^+$, found: 415.1253.

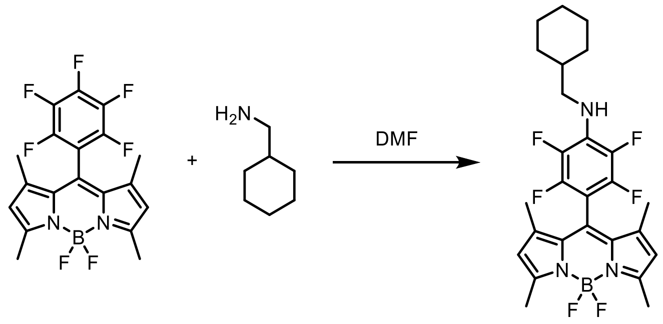

BDPF$_4$-cyH (4). 50 mg (0.12 mmol) of BDP-F$_5$ were dissolved in 5 mL of dimethylformamide and 130 μL (113 mg, 1.0 mmol) of cyclohexanemethylamine were added. After stirring for 3 h at rt, the reaction mixture was extracted with water/diethylether and the organic phase was dried over $MgSO_4$. After removal of the solvents, the crude product was purified on a column of neutral alumina (PE/DCM 3:1), eluting the product as second yellow-colored band (after some residual starting material). Yield: 39 mg (0.077 mmol, 64%) of a red solid. ^{1}H NMR 400 MHz, DMSO-d$_6$ δ (ppm) = 6.27 (s, 2H, Pyr-H), 3.03 (t, J = 6.6 Hz, 2H, Cy-CH_2-NH), 2.46 (s, 6H, Pyr-CH_3), 1.70-1.60 (m, 5H, cyH), 1.66 (s, 6H, Pyr-CH_3), 1.29- 1.01 (m, 6H, cyH). ^{13}C NMR (100 MHz, DMSO-d$_6$) δ (ppm) = 156.5, 141.9, 131.1, 124.9, 122.0, 49.5, 38.2, 30.1, 26.0, 25.3, 14.3, 12.9. HRMS-ESI: m/z:calcd for $C_{26}H_{29}BF_6N_3^+$: 508.2353 [M + H]$^+$, found: 508.2356.

3.5 Synthesis of BDP-AMADA (5)

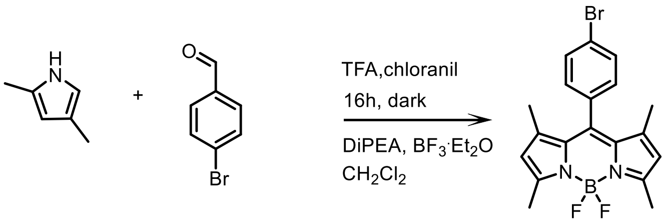

BDP-Br. 0.9 g (9.4 mmol) of 2,4-dimethylpyrrole and 0.87 g (4.7 mmol) of 4-bromobenzaldehyde were dissolved in 200 mL of dichloromethane and deoxygenated with Ar. Some drops of trifluoroacetic acid were added and the reaction mixture was stirred for 16 h in the dark. 1125 mg (4.65 mmol) of chloranil were added, followed by stirring for 2 h. Then, 4.9 mL of diisopropylethylamine were added and after 5 min of stirring, 5.2 mL of boron trifluoride diethyletherate were added, followed by stirring for 2 h. The reaction mixture was filtered through a pad of silica, eluted with dichloromethane and the solvents were evaporated to dryness. The crude product was purified on a silica column (PE/EtOAc 9:1) yielding 335 mg (0,83 mmol, 18%) of a red solid. ^{1}H NMR 400 MHz, DMSO-d_6 δ (ppm) = 7.77 (d, J = 8.6 Hz, 2H,Ar-H), 7.36 (d, J = 8.6 Hz, 2H, Ar-H), 6.20 (s, 2H, Pyr-H), 2.45 (s, 6H, Pyr-CH_3), 1.38 (s, 6H, Pyr-CH_3). ^{13}C NMR (100 MHz, DMSO-d_6) δ = 155.1, 142.5, 140,3, 133.1, 132.2, 130.4, 130.1, 122.5, 121.4, 14.1, 14.0. HRMS-ESI: m/z:calcd for $C_{19}H_{19}BBrF_2N_2^+$: 403.0787 [M + H]+, found: 403.0793.

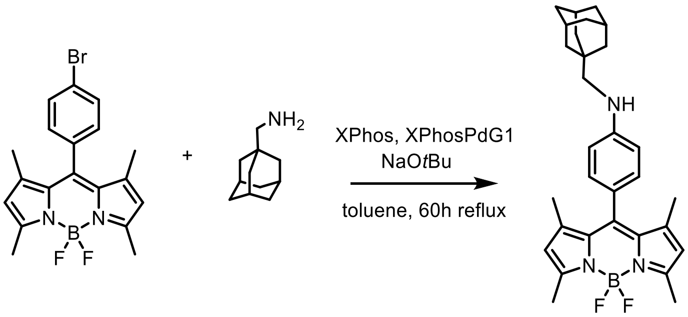

BDP-AMADA (5). Under an argon atmosphere, 60.2 mg (0.15 mmol) of BDP-Br, 16.2 mg (0.034 mmol) of 2-dicyclohexylphosphino-2′,4′,6′-triisopropylbiphenyl (XPhos), 23.6 mg (0.032 mmol) of (2-dicyclohexylphosphino-2′,4′,6′-triisopropyl-1,1′-biphenyl) [2-(2-aminoethyl)phenyl)]palladium(II) chloride (XPhos Pd G1) and 29 mg (0.30 mmol) of sodium *tert*-butoxide were added to 6 mL of dry toluene. Subsequently, 28 μL (26 mg, 0.23 mmol) of 1-adamantanemethylamine were added. The reaction mixture was heated under reflux for 40 h and after cooling to rt filtered through a pad of silica. The crude product was eluted with DCM and evaporated to dryness. After purification on a neutral alumina column (PE/DCM 3:1 with increasing amounts of DCM), the product was eluted as second, non-fluorescent yellow-colored fraction (after some residual BDP-Br starting material) in 12% yield (9 mg, 0.018 mmol). ^{1}H NMR 400 MHz, $CDCl_3$ δ (ppm) = 7.32 (d, J = 8.8 Hz, 2H, Ar-H), 7.21 (d, J = 8.8 Hz, 2H, Ar-H), 5.99 (s, 2H, Pyr-H), 2.58-2.54 (m, 8H, Cy-CH_2-NH, Pyr-CH_3), 1.78-1.60 (m, 3H, ADAMA), 1.51 (s, 6H, Pyr-CH_3; (m, 2H, ADAMA)), 1.29-1.57 (m, 5H, ADAMA), 0.94-0.82 (m, 4H, ADAMA). ^{13}C NMR (100 MHz, $CDCl_3$) δ = 155.2, 146.2, 144.6, 131.5, 128.8, 127.1, 126.5, 125.2, 121.1, 119.6, 117.2, 108.5, 51.9, 40.8, 37.1, 29.7, 28.4, 28.1, 14.7, 14.6, 14.5. HRMS-ESI: m/z:calcd for $C_{30}H_{37}BF_2N_3^+$: 488.3043 [M + H]+, found: 488.3078.

3.6 NMR Spectra of Synthesized Compounds

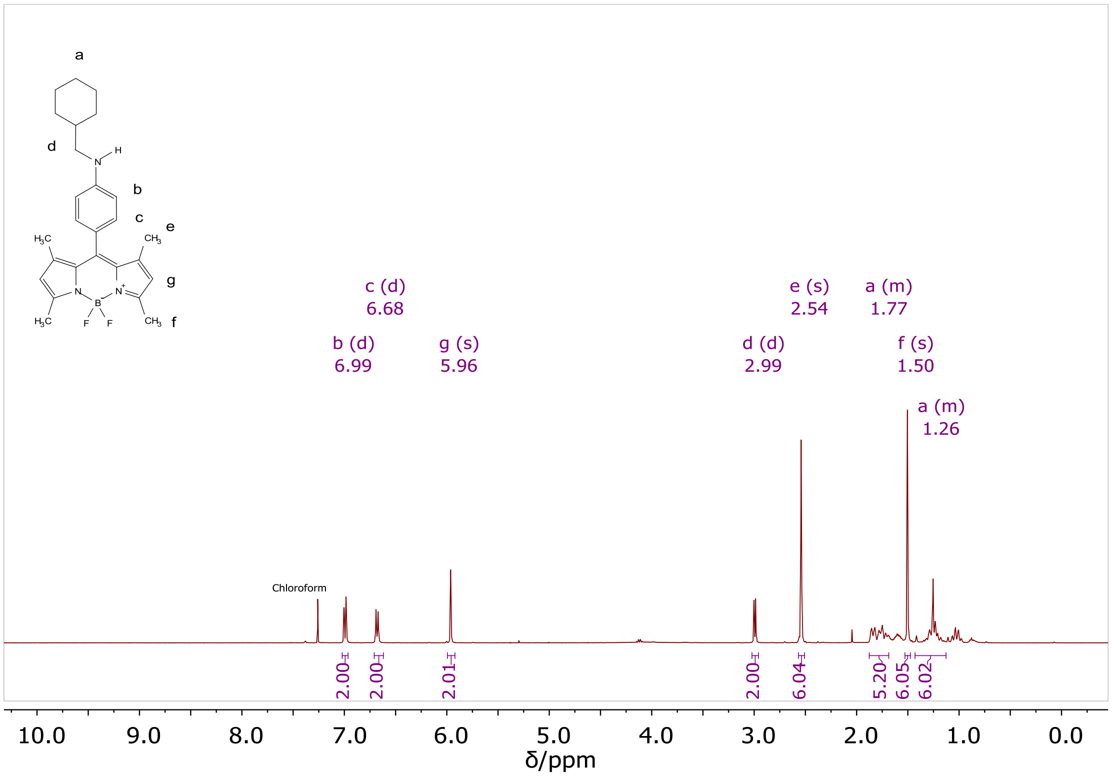

Figure S1: ^{1}H NMR spectrum of **2**.

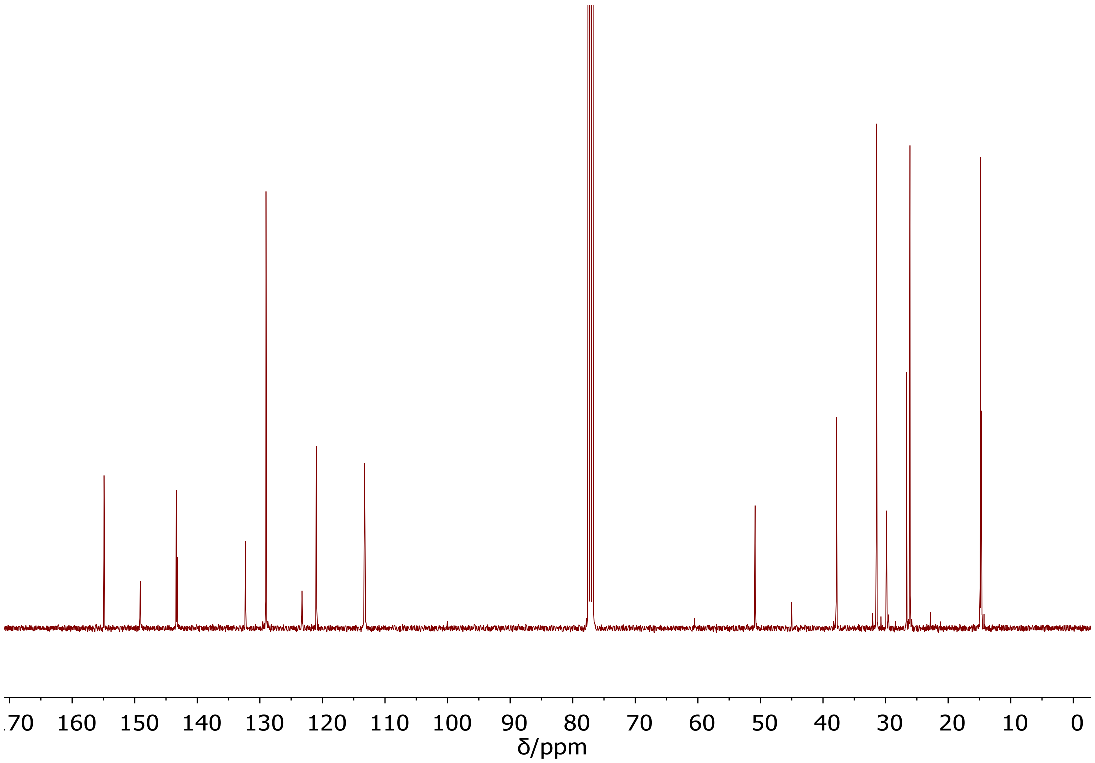

Figure S2: ^{13}C NMR spectrum of **2**.

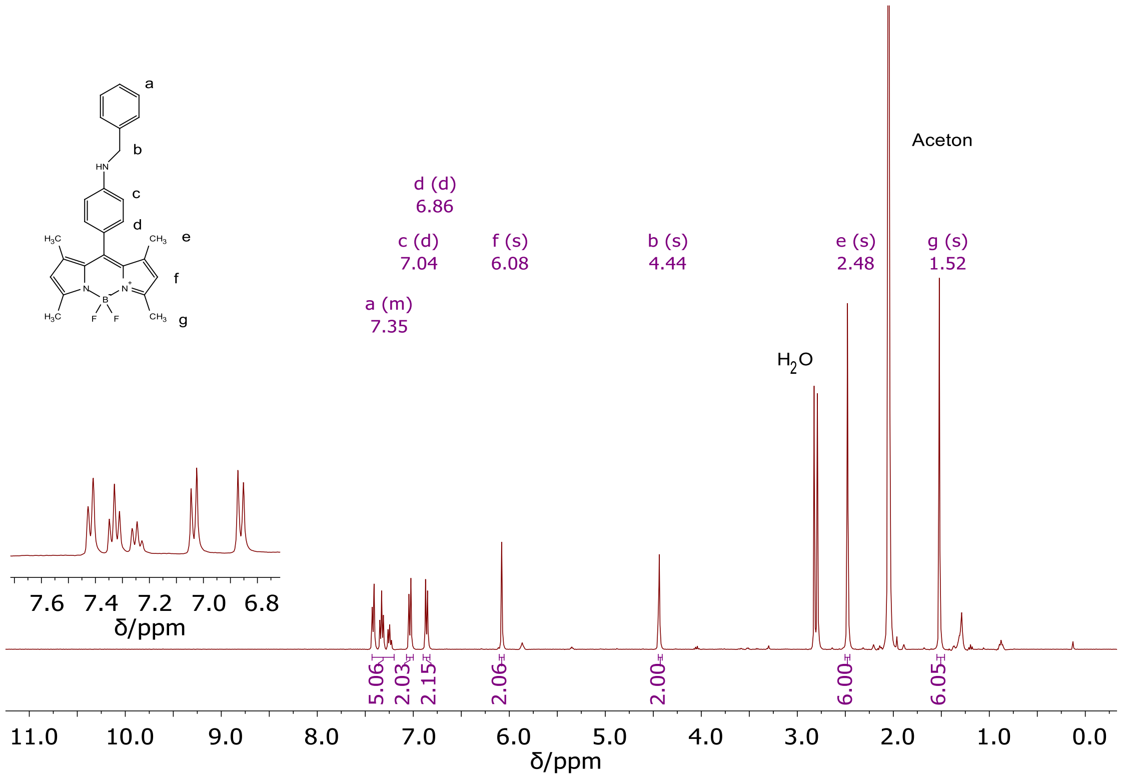

Figure S3: ^{1}H NMR spectrum of **1**.

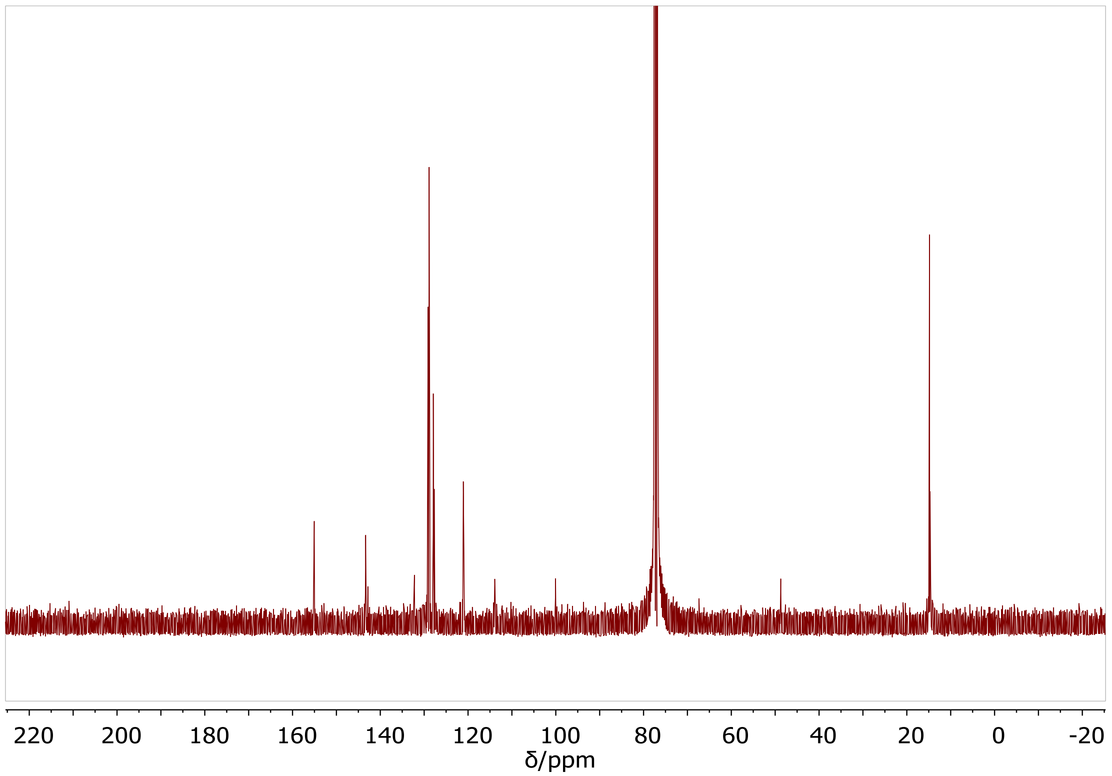

Figure S4: ^{13}C NMR spectrum of **1**.

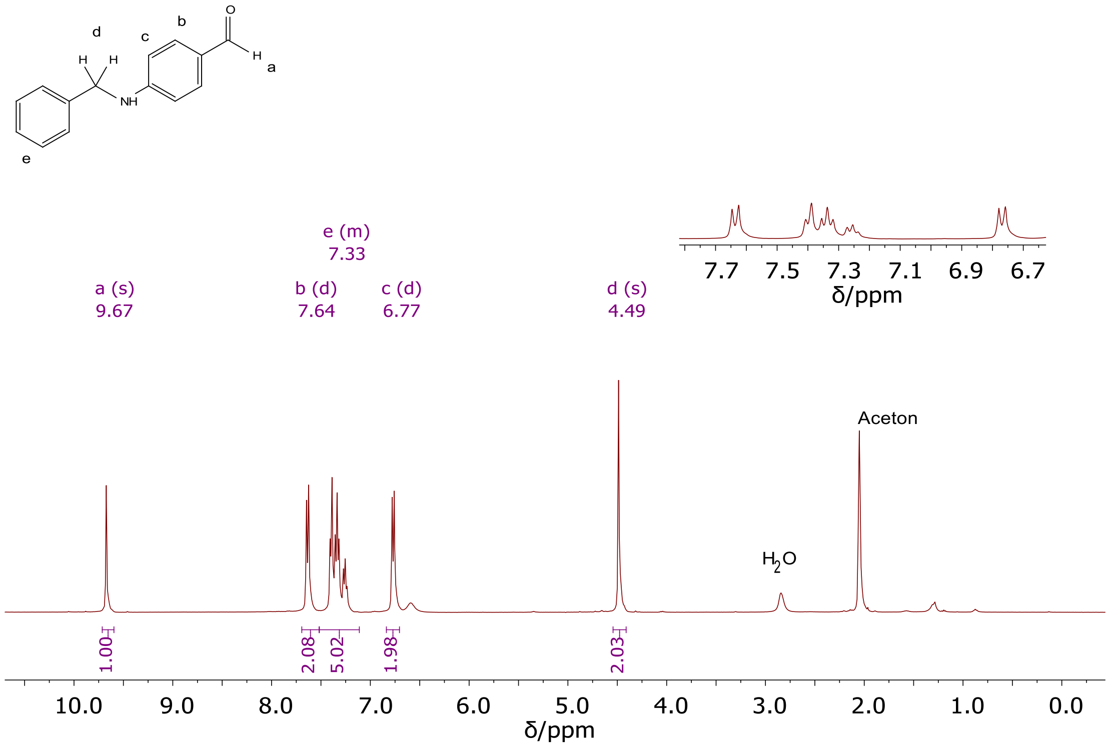

Figure S5: ^{1}H NMR spectrum of *p*-(benzylamino)benzaldehyde.

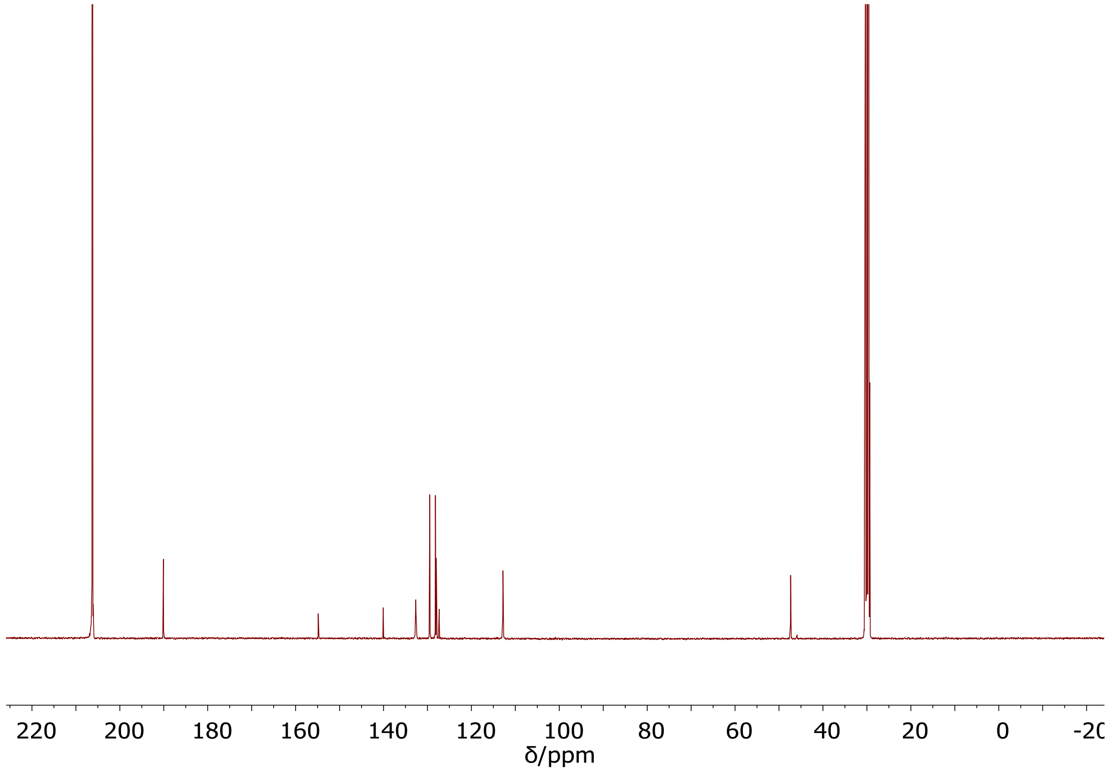

Figure S6: ^{13}C NMR spectrum of *p*-(benzylamino)benzaldehyde.

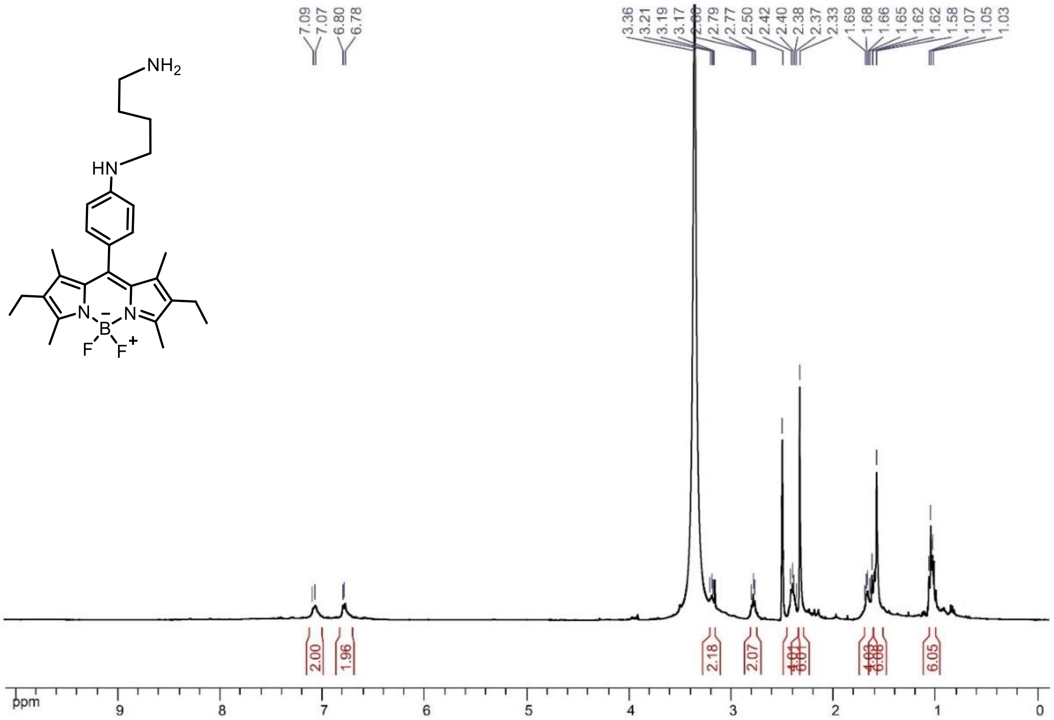

Figure S7: ^{1}H NMR spectrum of **3**.

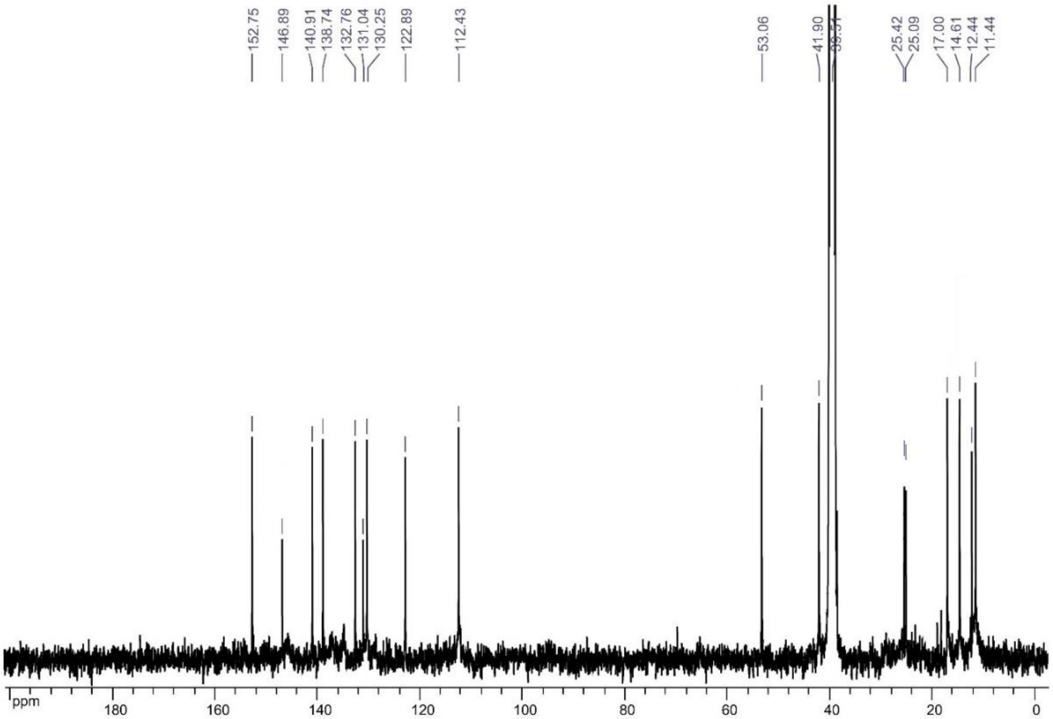

Figure S8: ^{13}C NMR spectrum of **3**.

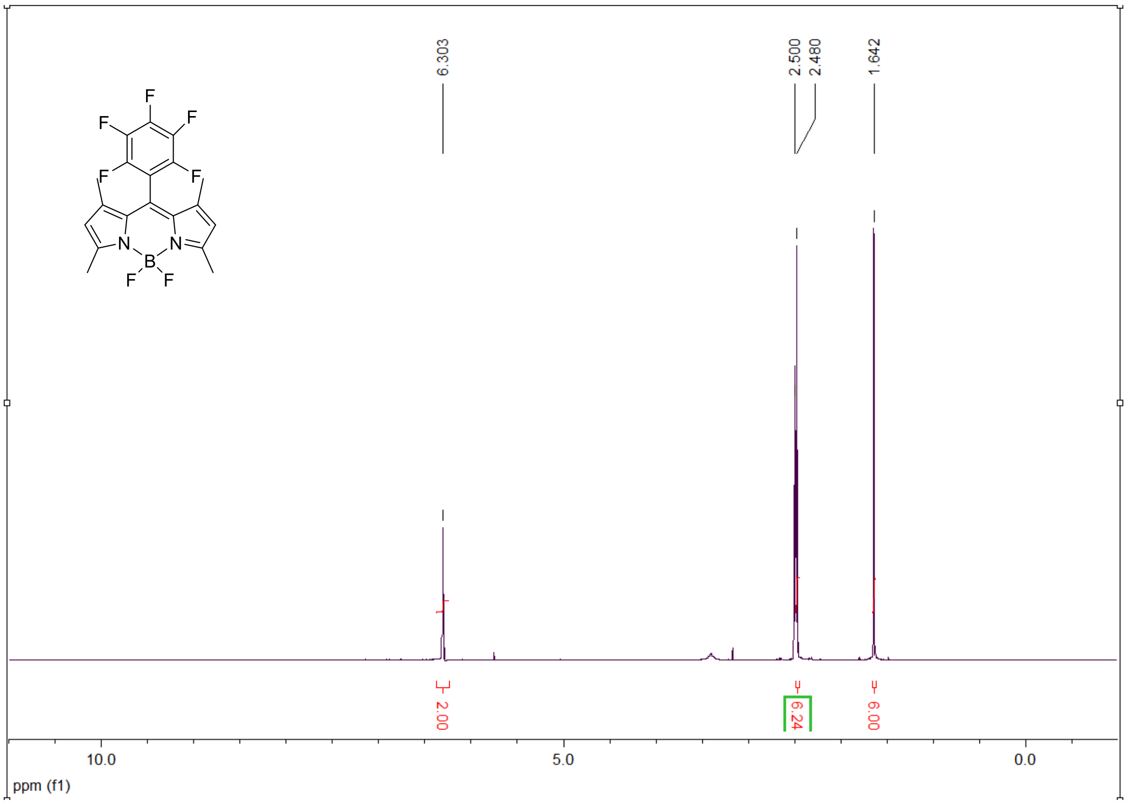

Figure S9: ^{1}H NMR spectrum of **BDP-F5.**

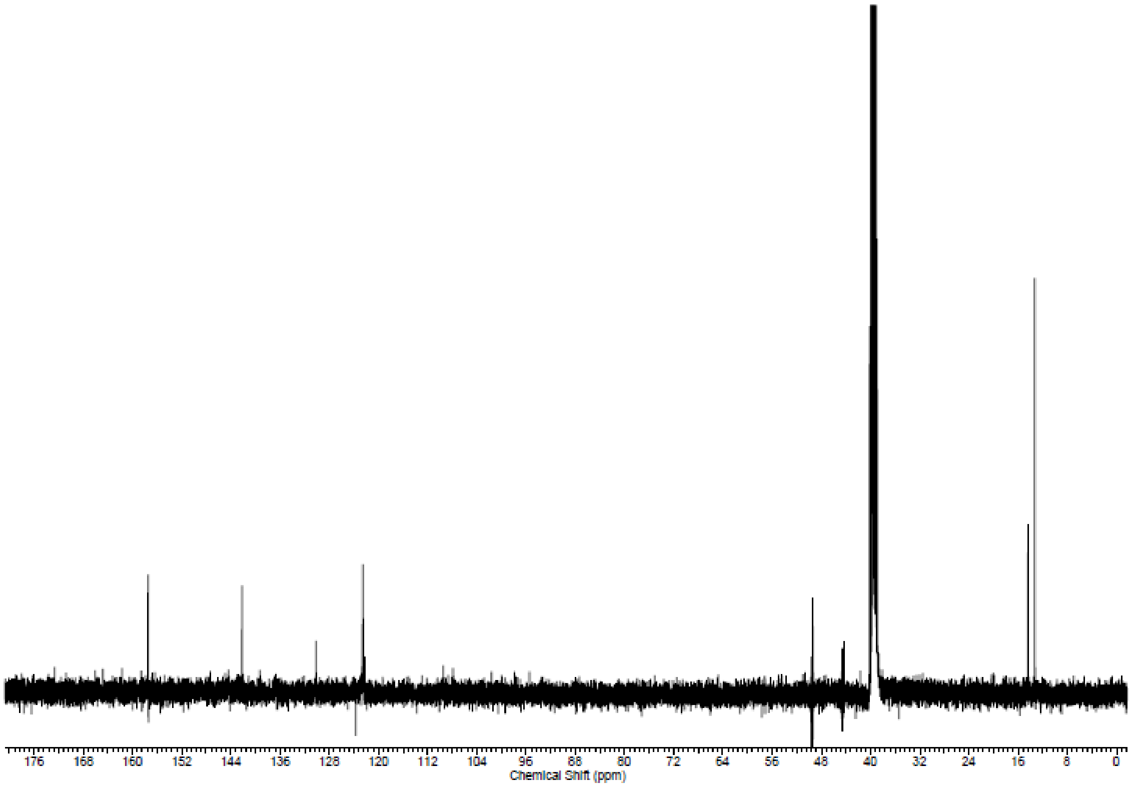

Figure S10: ^{13}C NMR spectrum of **BDP-F5.**

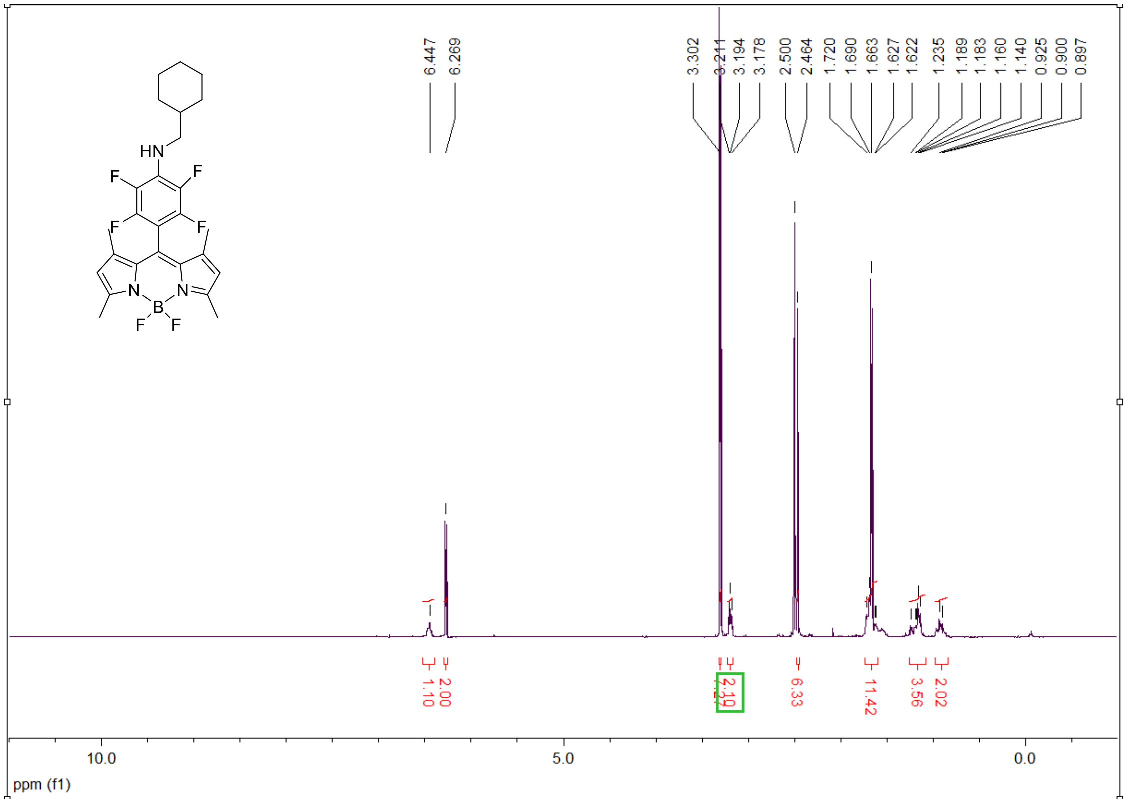

Figure S11: ^{1}H NMR spectrum of **4.**

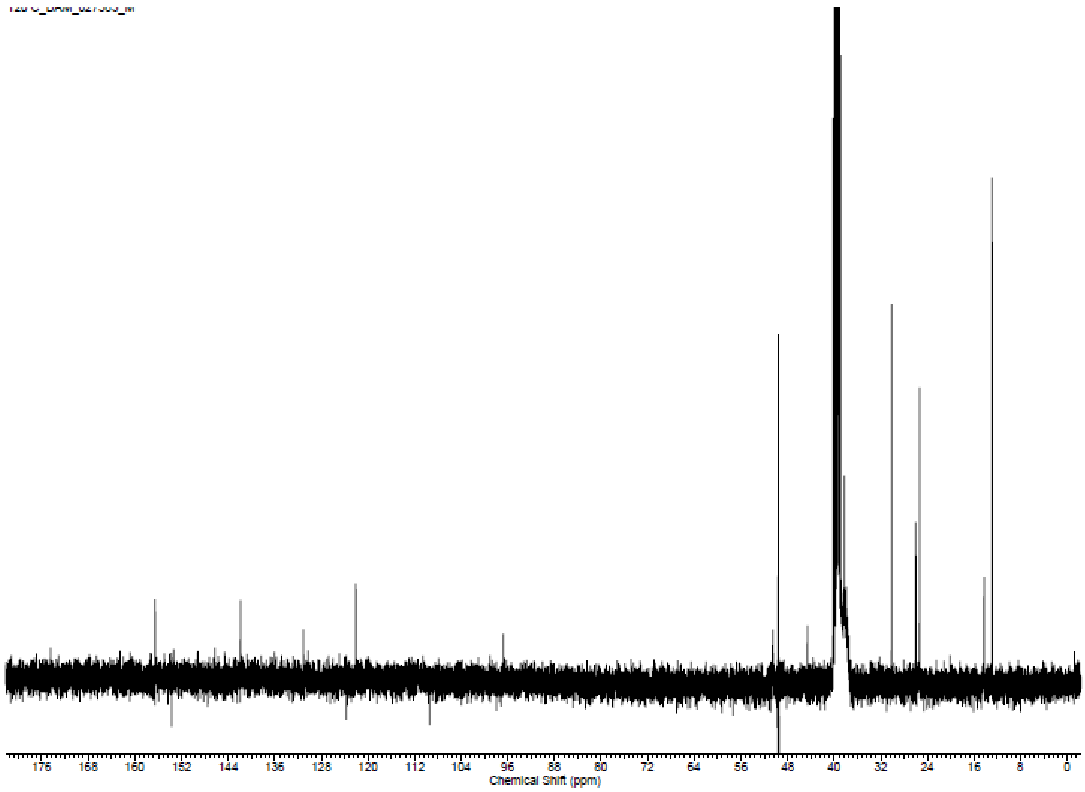

Figure S12: ^{13}C NMR spectrum of **4.**

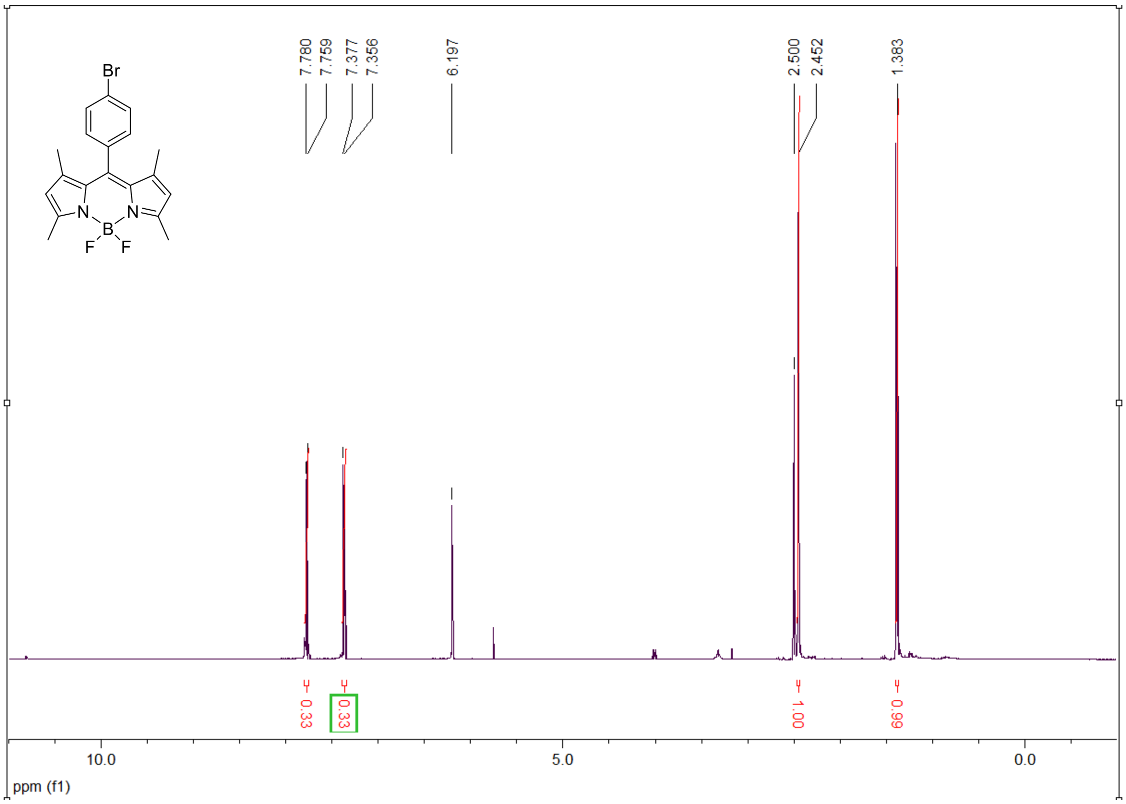

Figure S13: ^{1}H NMR spectrum of **BDP-Br**.

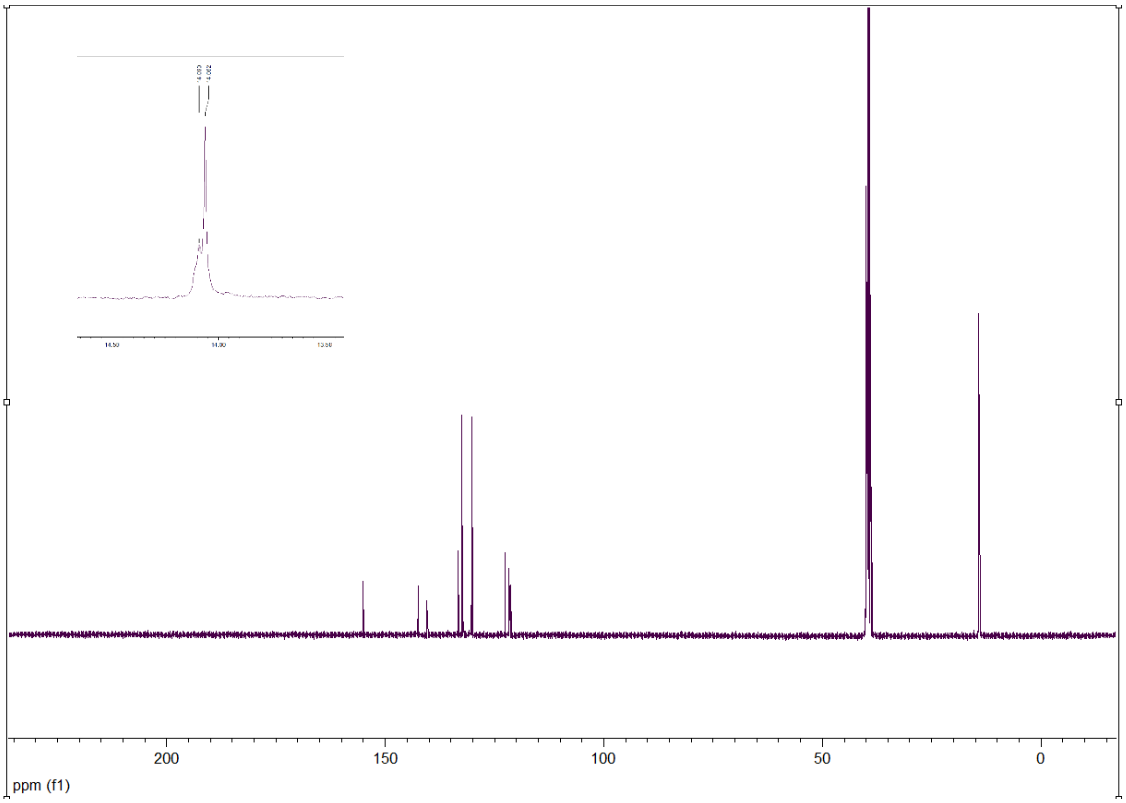

Figure S14: ^{13}C NMR spectrum of **BDP-Br**. Inset: 2 Peaks at around 14 ppm**.**

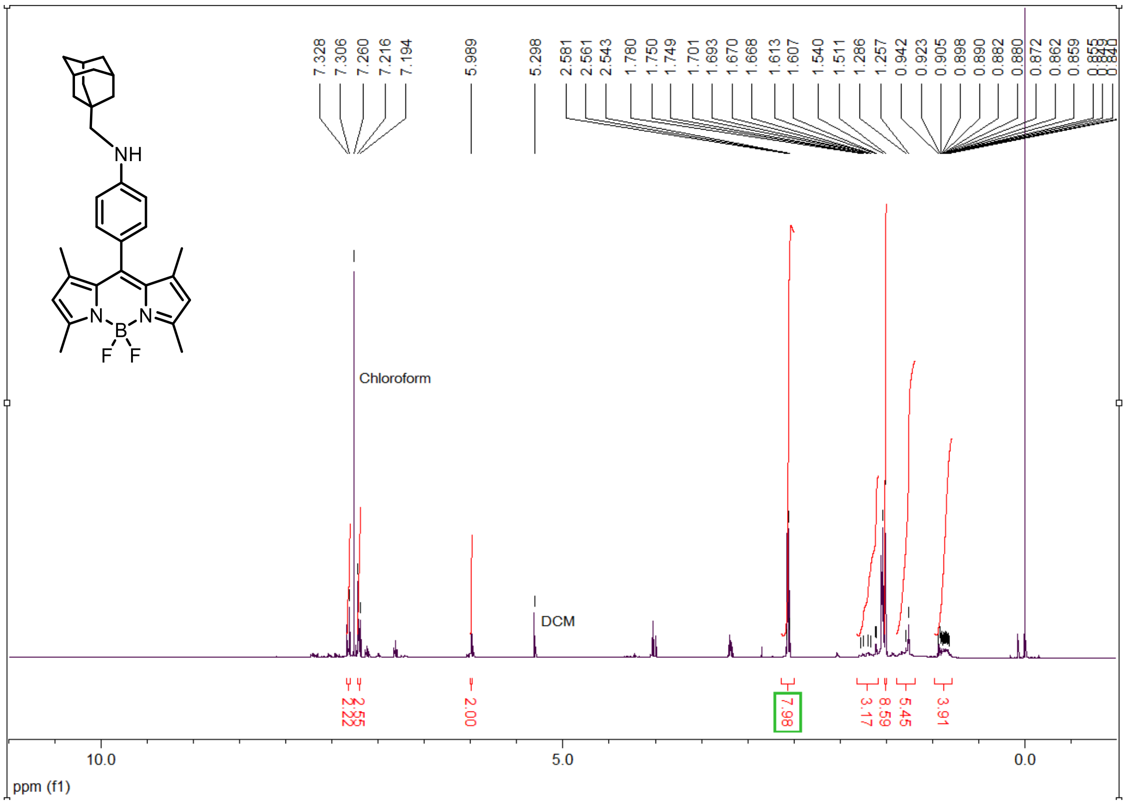

Figure S15: ^{1}H NMR spectrum of **5**.

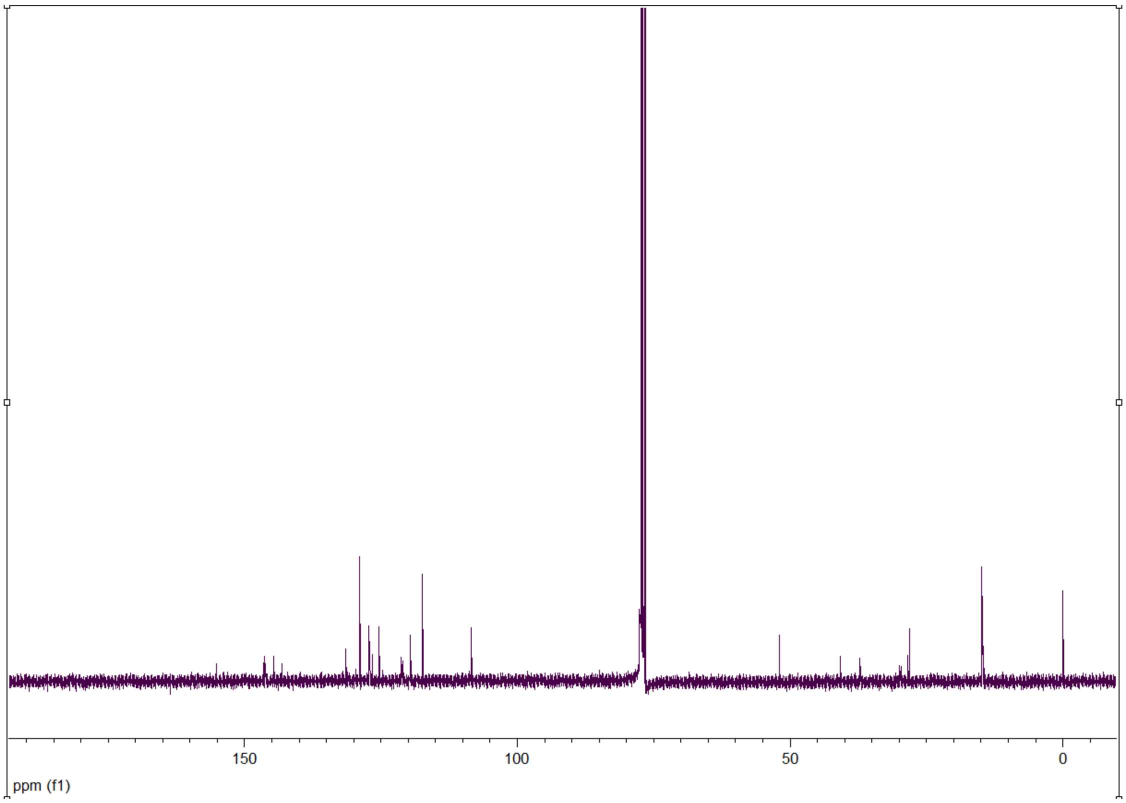

Figure S16: ^{13}C NMR spectrum of **5**.

4 DETERMINATION OF FLUORESCENCE QUANTUM YIELDS

Quantum yields were determined according to established literature methods [8]. In brief, the integrated area of the fluorescence emission bands of the BODIPY samples, I_{BDP}, and the standard, I_{St}, their respective absorbance at the excitation wavelength, A_{BDP} and A_{St}, and the refractive index of the solvents, η_{BDP} and η_{St}, were used in conjunction with the fluorescence quantum yield of the standard, Φ_{St}, to calculate the quantum yields of the BODIPYs, Φ_{BDP}, according to the following equation:

$$\phi_{BDP} = \phi_{\mathrm{St}} \times \frac{I_{\mathrm{BDP}}\, A_{\mathrm{St}}}{I_{\mathrm{St}}\, A_{\mathrm{BDP}}} \times \frac{\eta_{\mathrm{BDP}}^2}{\eta_{\mathrm{St}}^2}$$

Specifically, fluorescein in 0.1 M NaOH was taken as standard (Φ_{St} = 0.89) and the refractive index of the ACN/water mixtures was calculated by considering a linear dependence of the refractive index on the mole fraction of the solvent mixture [9]. Correction curves for the fluorescence measurements were generated with a dye kit certified by Federal Institute for Materials Research and Testing (BAM, Germany) [2-6].

5 GLOBAL FITTING PROCEDURE

Binding between CB7 and protonated as well as deprotonated BODIPY dyes is described by the following thermodynamic cycle (also see Figure 1a in main text):

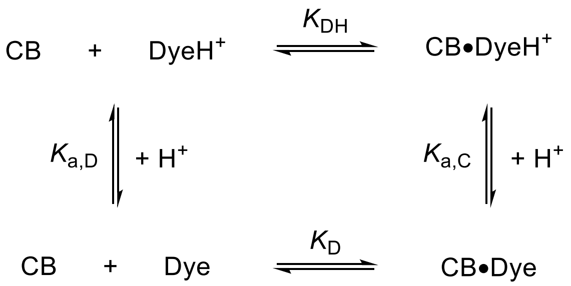

From the law of mass action, it follows:

$$K_{\mathrm{D}} = \frac{[\mathrm{CB \bullet Dye}]}{[\mathrm{CB}][\mathrm{Dye}]} \quad \text{[eq1]}$$

$$K_{\mathrm{DH}} = \frac{[\mathrm{CB \bullet DyeH^+}]}{[\mathrm{CB}][\mathrm{DyeH^+}]} \quad \text{[eq2]}$$

$$K_{\mathrm{a,D}} = \frac{[\mathrm{Dye}][\mathrm{H^+}]}{[\mathrm{DyeH^+}]} \quad \text{[eq3]}$$

$$K_{\mathrm{a,C}} = \frac{[\mathrm{CB \bullet Dye}][\mathrm{H^+}]}{[\mathrm{CB \bullet DyeH^+}]} \quad \text{[eq4]}$$

Law of conservation of mass requires for the total concentrations of CB, $[\mathrm{CB}]_0$, and BODIPY dye, $[\mathrm{Dye}]_0$, that:

$$[\mathrm{CB \bullet Dye}] + [\mathrm{CB \bullet DyeH^+}] = [\mathrm{CB}]_0 - [\mathrm{CB}] \quad \text{[eq5]}$$

$$[\mathrm{CB \bullet Dye}] + [\mathrm{CB \bullet DyeH^+}] = [\mathrm{Dye}]_0 - [\mathrm{Dye}] - [\mathrm{DyeH^+}] \quad \text{[eq6]}$$

Combining eq5 with eq1 to and eq4 affords:

$$\frac{K_{\mathrm{a,C}}[\mathrm{CB \bullet DyeH^+}]}{[\mathrm{H^+}]} + [\mathrm{CB \bullet DyeH^+}] = [\mathrm{CB}]_0 - \frac{K_{\mathrm{a,C}}[\mathrm{CB \bullet DyeH^+}]}{K_{\mathrm{D}}[\mathrm{Dye}][\mathrm{H^+}]} \quad \text{[eq7]}$$

Combining eq6 with eq3 and eq4 affords:

$$\frac{K_{\mathrm{a,C}}[\mathrm{CB \bullet DyeH^+}]}{[\mathrm{H^+}]} + [\mathrm{CB \bullet DyeH^+}] = [\mathrm{Dye}]_0 - [\mathrm{Dye}]\left(1 + \frac{[\mathrm{H^+}]}{K_{\mathrm{a,D}}}\right) \quad \text{[eq8]}$$

Equations eq7 and eq8 can now be solved for [Dye] and combined to afford:

$$0 = a[\mathrm{CB{\bullet}DyeH^+}]^2 + b[\mathrm{CB{\bullet}DyeH^+}] + c \qquad \text{[eq9]}$$

where

$$a = \frac{{K_{\mathrm{a,C}}}^2}{[\mathrm{H^+}]} + 2K_{\mathrm{a,C}} + [\mathrm{H^+}]$$

$$b = -\left(\left(K_{\mathrm{a,C}} + [\mathrm{H^+}]\right)[\mathrm{Dye}]_0 + \left(K_{\mathrm{a,C}} + [\mathrm{H^+}]\right)[\mathrm{CB}]_0 + \frac{K_{\mathrm{a,C}}K_{\mathrm{a,D}} + K_{\mathrm{a,C}}[\mathrm{H^+}]}{K_{\mathrm{a,D}}K_{\mathrm{D}}}\right)$$

$$c = [\mathrm{Dye}]_0[\mathrm{H^+}][\mathrm{CB}]_0$$

Eq9 can be solved analytically to afford $[\mathrm{CB{\bullet}DyeH^+}]$.

The fluorescence intensity is obtained from the mole fraction of each species (unprotonated dye D and complex DCB as well as protonated dye DH+ and complex DCBH+) and their respective intensities at the applied concentration:

$$FI = \frac{[\mathrm{Dye}]}{[\mathrm{Dye}]_0}I_D + \frac{[\mathrm{CB{\bullet}Dye}]}{[\mathrm{Dye}]_0}I_{DCB} + \frac{[\mathrm{DyeH^+}]}{[\mathrm{Dye}]_0}I_{DH+} + \frac{[\mathrm{CB{\bullet}DyeH^+}]}{[\mathrm{Dye}]_0}I_{DCBH+} \qquad \text{[eq10]}$$

In our case, the fluorescence is only affected by protonation and not by complexation such that $I_D = I_{DCB}$ and $I_{DH+} = I_{DCBH+}$. Eq10 can thus be simplified and combined with eq 3 and eq8 to afford:

$$FI = I_D + (I_{DH+} - I_D)\left(\frac{[\mathrm{Dye}]_0[\mathrm{H^+}] - K_{\mathrm{a,C}}[\mathrm{CB{\bullet}DyeH^+}] - [\mathrm{CB{\bullet}DyeH^+}][\mathrm{H^+}]}{\left(K_{\mathrm{a,D}} + [\mathrm{H^+}]\right)[\mathrm{Dye}]_0} + \frac{[\mathrm{CB{\bullet}DyeH^+}]}{[\mathrm{Dye}]_0}\right) \qquad \text{[eq11]}$$

Eq11 and eq9 were then implemented into Origin 9 and titration data at various pH (see for example Figure 3b in main text) was simultaneously analyzed using the Global Fitting procedure from Origin 9.

6 SUPPORTING FIGURES

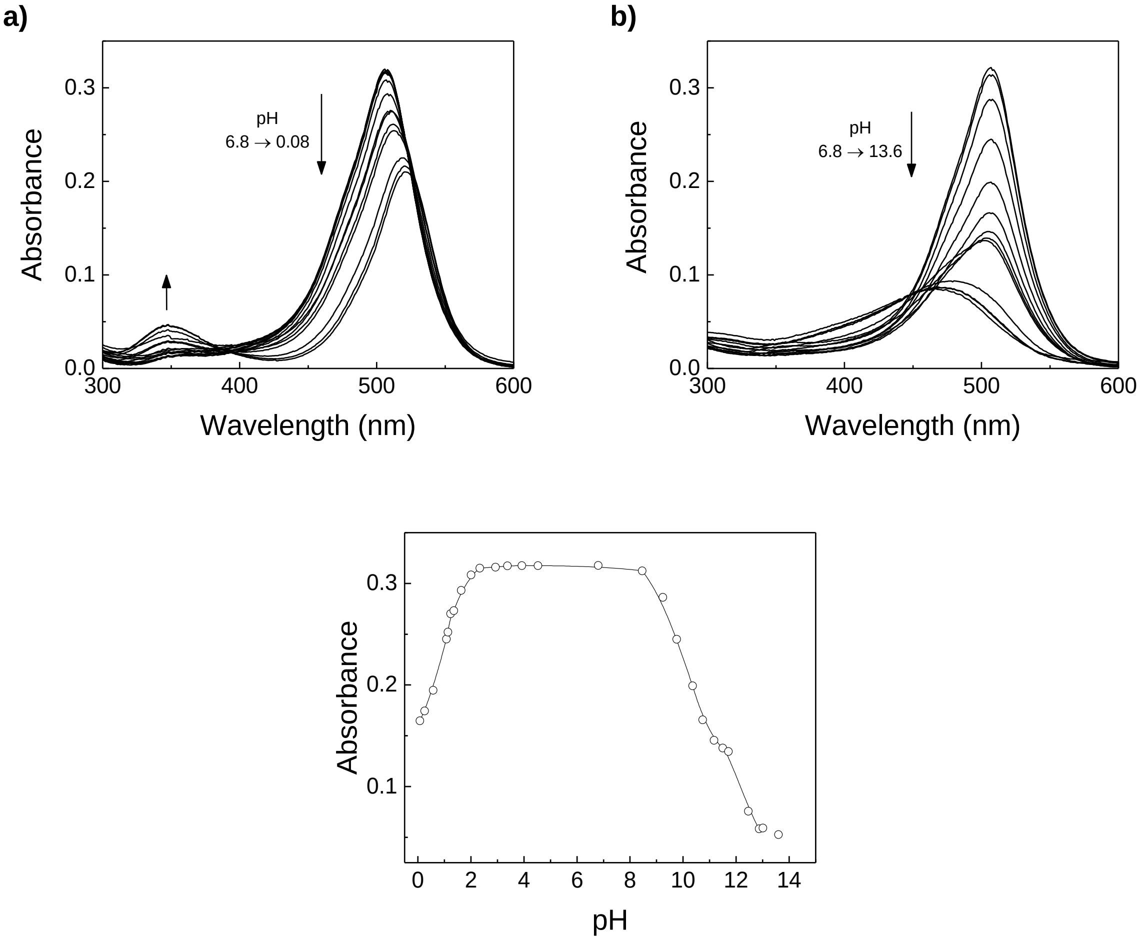

Figure S17: Absorption spectral changes with varying pH for **3**. H_2O/ACN 98:2, λ_{obs} = 506 nm.

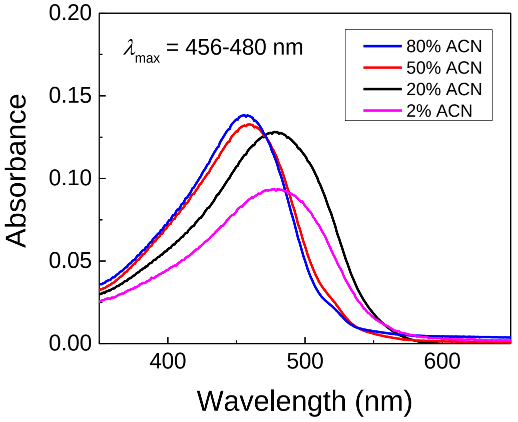

Figure S18: Solvatochromism of 10 µM **3** in varying H_2O/ACN mixtures at pH 12.0.

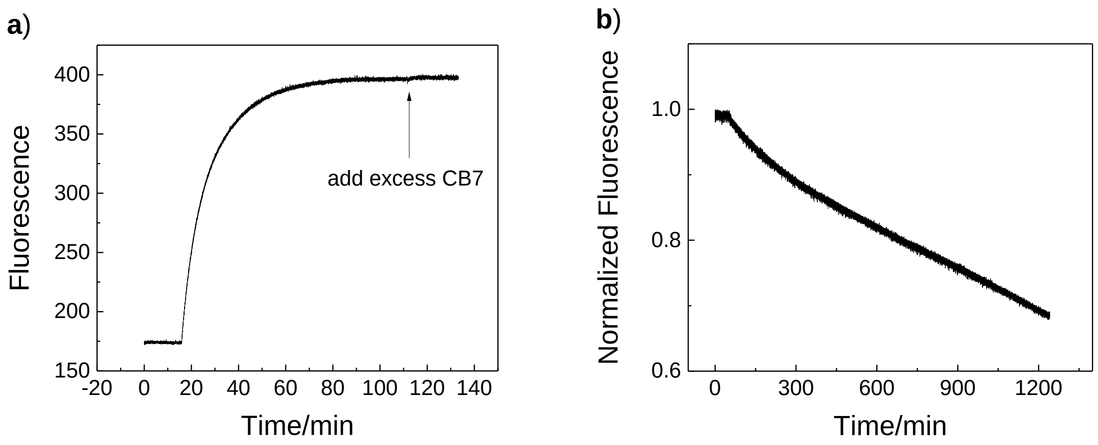

Figure S19: Fluorescence association and dissociation kinetic for 200 nM **5** in 10 mM citrate buffer, pH 3.33 (30% (v/v) ACN in water). a) Association kinetics with 200 nM CB7. b) Competitive dissociation kinetics of 200 nM **5**●CB7 complex with 1.5 mM adamantylamine as a competitor.

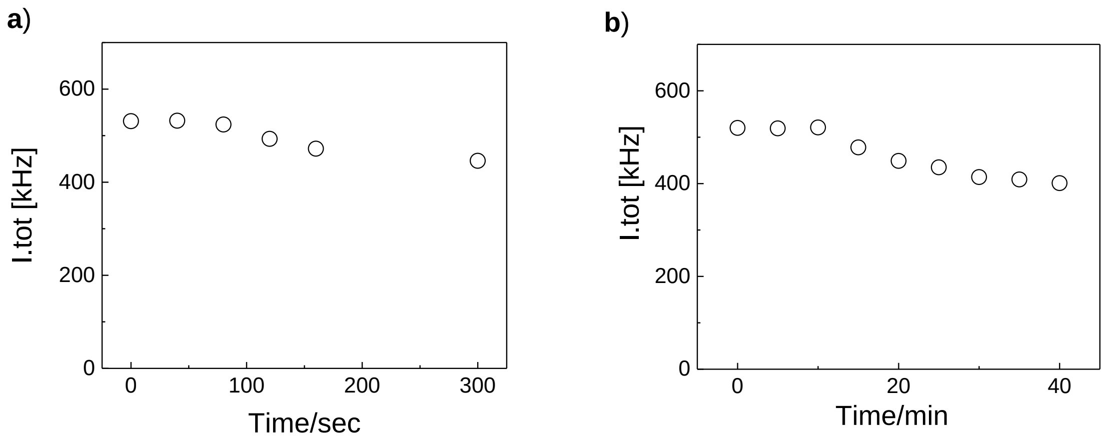

Figure S20: Photostability measurements by FCS with time for 10 nM of **2** at pH 1.5 (30% (v/v) ACN in water) a) in absence and b) presence of 100 μM CB7.

7 REFERENCES

1. Marquez, C.; Fang, H.; Nau, W. M. *IEEE Trans. Nanobiosci.* **2004**, *3*, 39-45.
2. Resch-Genger, U.; Pfeifer, D.; Monte, C.; Pilz, W.; Hoffmann, A.; Spieles, M.; Rurack, K.; Hollandt, J.; Taubert, D.; Schönenberger, B.; Nording, P. *J. Fluoresc.* **2005**, *15*, 315-336.
3. Resch-Genger, U.; Hoffmann, K.; Nietfeld, W.; Engel, A.; Neukammer, J.; Nitschke, R.; Ebert, B.; Macdonald, R. *J. Fluoresc.* **2005**, *15*, 337-362.
4. Hoffmann, K.; Resch-Genger, U.; Mix, R.; Friedrich, J. F. *J. Fluoresc.* **2006**, *16*, 441-448.
5. Monte, C.; Pilz, W.; Resch-Genger, U. In *Proc. SPIE Conf.*; SPIE, 2005, pp 1-10.
6. Resch-Genger, U.; Hoffmann, K.; Hoffmann, A. *Ann. N. Y. Acad. Sci.* **2008**, *1130*, 35-43.
7. Zhao, T.; Kurpiewska, K.; Kalinowska-Tluscik, J.; Herdtweck, E.; Domling, A. *Chem. Eur. J.* **2016**, *22*, 3009-3018.
8. Würth, C.; Grabolle, M.; Pauli, J.; Spieles, M.; Resch-Genger, U. *Nat. Protoc.* **2013**, *8*, 1535-1550.
9. Bertie, J. E.; Lan, Z. *J. Phys. Chem. B* **1997**, *101*, 4111-4119.
